# Iron-Catalyzed Cross-Coupling of *Bis*-(aryl)manganese Nucleophiles with Alkenyl Halides: Optimization and Mechanistic Investigations

**DOI:** 10.3390/molecules25030723

**Published:** 2020-02-07

**Authors:** Lidie Rousseau, Alexandre Desaintjean, Paul Knochel, Guillaume Lefèvre

**Affiliations:** 1 Chimie ParisTech, PSL University, CNRS, Institute of Chemistry for Life and Health Sciences (i-CLeHS FRE2027), CSB2D, 75005 Paris, France; lidie.rousseau@cea.fr; 2 NIMBE, CEA, CNRS, Univ. Paris-Saclay, 91191 Gif-sur-Yvette, France; 3 Department of Chemistry, Ludwig-Maximilians-Universitat München, Butenandstr. 5-13, Haus F, 81377 Munich, Germany; Alexandre.Desaintjean@cup.uni-muenchen.de (A.D.); Paul.Knochel@cup.uni-muenchen.de (P.K.)

**Keywords:** iron catalysis, cross-coupling, *bis*-(aryl)manganese, alkenyl halides, *ate* iron(II) complex

## Abstract

Various substituted *bis*-(aryl)manganese species were prepared from aryl bromides by one-pot insertion of magnesium turnings in the presence of LiCl and in situ trans-metalation with MnCl_2_ in THF at −5 °C within 2 h. These *bis*-(aryl)manganese reagents undergo smooth iron-catalyzed cross-couplings using 10 mol% Fe(acac)_3_ with various functionalized alkenyl iodides and bromides in 1 h at 25 °C. The aryl-alkenyl cross-coupling reaction mechanism was thoroughly investigated through paramagnetic ^1^H-NMR, which identified the key role of *tris*-coordinated *ate*-iron(II) species in the catalytic process.

## 1. Introduction

Transition-metal catalyzed cross-couplings are widely used in the development and production of pharmaceutical compounds [1]. The most versatile of them are palladium-catalyzed and nickel-catalyzed cross-couplings [2,3,4,5] as they tolerate a great variety of functionalities on both coupling partners. Yet, these metals have drawbacks such as toxicity [6,7] and high prices in the case of palladium [8]. That is one of the reasons why copper [9], iron [10,11,12,13], or cobalt [14] have been developed as alternative metal-catalysts.

Organomanganese species pioneered by Cahiez [15] often considerably reduce the amount of side reactions such as homo-coupling [16,17] and have proven to be excellent nucleophiles in various types of reactions [18,19,20,21,22,23,24] including cross-couplings [25,26]. Organomanganese species then constitute an interesting alternative to usual cross-coupling partners such as organomagnesium [27], organozinc [28], and organo-boronic esters, which may have genotoxic properties [29,30].

**Scheme 1:**
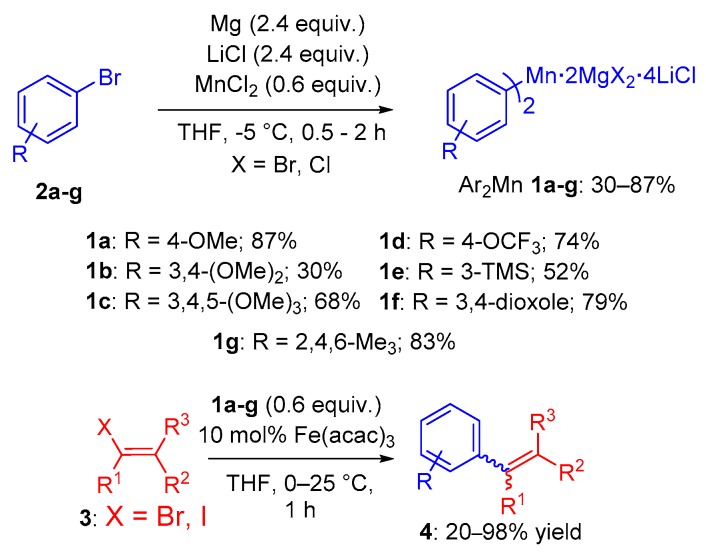
One-pot preparation of *bis*-(aryl)manganese reagents by in situ trans-metalation followed by iron-catalyzed cross-couplings with alkenyl iodides and bromides.

Recently, we have developed a two-step preparation of functionalized *bis*-(aryl)manganese reagents by oxidative insertion of magnesium into the C-Br bond of aryl bromides, which is followed by a trans-metalation with MnCl_2_·2LiCl [31]. Herein, we wish to report an effective one-pot preparation of those functionalized *bis*-(aryl)manganese reagents (Ar_2_Mn•2MgX_2_•4LiCl, denoted as Ar_2_Mn (1), Scheme 1) starting from aryl bromides, which are followed by an iron-catalyzed cross-coupling of 1 with alkenyl iodides and bromides, and provide a range of polyfunctionalized alkenes (4, Scheme 1). These *bis*-(aryl)manganese reagents are generally stable at RT (25 °C) for several hours, which makes them suitable reagents for mild cross-coupling reactions [32].

## 2. Results

In preliminary experiments, the *bis*-(aryl)manganese reagent **1a** was conveniently prepared by treating 4-bromoanisole (**2a**, 1.0 equiv.) in THF at −5 °C with magnesium turnings and LiCl (2.4 equiv.) in the presence of MnCl_2_ (0.6 equiv.) within 1 h. Titration [31] with iodine led to a yield for **1a** of 87%. Cross-coupling methodologies involving those *bis*-(aryl)manganese species were then investigated (see Supporting Information file for details).

In the absence of any iron catalyst, the cross-coupling of **1a** with (*Z*)-ethyl 3-iodoacrylate (**3a**; 25 °C, 1 h) produced a *Z*/*E* = 50:50 mixture of the desired cross-coupling product **4a** in 60% yield (Table 1, entry 1). Although the cross-coupling performed with FeBr_2_ gave a moderate yield of 42%, the use of FeCl_3_, FeBr_3_, or FeCl_2_ afforded the *E* isomer of **4a** in 54–64% yield (entries 2–5). Using Fe(acac)_2_ proved to be more effective since the yield increased to 67% (entry 6). Our best result was obtained with Fe(acac)_3_ (>99% purity) as a catalyst, producing the *E* isomer of **4a** in a 79% yield (entry 7).

Furthermore, the cross-coupling of 1a with (2-bromovinyl)trimethylsilane (**3b**; *Z*/*E* = 10:90) gave the olefin **4b** in 98% yield with complete *E*-selectivity (*Z*/*E* = 1:99) whereas the yield without iron salt was 24% (*Z*/*E* = 20:80, Table 2, entry 1). When the electron-rich *bis*-(3,4-dimethoxyphenyl)manganese (**1b**) was mixed with **3a**, the *E*-acrylate **4c** was generated in 69% yield and a *Z*/*E* = 69:31 mixture of products was obtained in 58% yield without an iron catalyst (entry 2). The tri-substituted *bis*-(3,4,5-trimethoxyphenyl)manganese (**1c**) underwent smooth cross-coupling with **3b** to afford the *E*-alkene **4d** in 80% (8% were obtained without a catalyst, entry 3). Additionally, **1c** reacted with **3a** and 2-bromostyrene (**3c**; *Z*/*E* = 18:82) to give the acrylate **4e** and **4f** (*Z*/*E* = 1:99) in 57% and 82% yield whereas 66% (*Z*/*E* = 72:28) and 80% (*Z*/*E* = 53:47) were, respectively, obtained without a catalyst (entries 4–5). In the last experiment, **1c** reacted with (*E*)-1-iodooctene (**3d**) to provide the alkene **4g** in 87% yield (*Z*/*E* = 9:91) when 77% yield (*Z*/*E* = 4:96) was obtained without a catalyst (entry 6). Furthermore, *bis*-(4-(trifluoromethoxy)phenyl)manganese (**1d**) reacted with **3a** to provide the acrylate **4h** (*Z*/*E* = 1:99) in 77% yield (entry 7). The reaction without Fe(acac)_3_ gave a similar yield but a mix of the two isomers (74%, *Z*/*E* = 81:19, entry 7). The silicon-containing *bis*-(aryl)manganese reagent **1e** could also react with **3a**, which produces **4i** (*Z*/*E* = 1:99) in 64% yield (51%, *Z*/*E* = 57:43 were obtained without Fe(acac)_3_, entry 8). Some good yields could be achieved in the absence of the iron catalyst (entries 2, 4–8), which could be attributed to the catalytic activity of the manganese(II) itself. For example, manganese salts proved to efficiently catalyze several couplings of organomagnesium reagents with alkenyl electrophiles in the past [32]. The *bis*-benzo[d][1,3]dioxol-5-ylmanganese (**1f**) also reacted with **3a** and **3b** to yield the *E*-alkenes **4j** and **4k** in 78–84% yield (entries 9–10). The bulkier *bis*-mesitylmanganese **1g** reacted with **3b** to afford **4l** with a small 20% yield (91% after 18 h, entry 11). This method also proved to tolerate nitriles, since 4-(2-bromovinyl)benzonitrile **3e** (*Z*/*E* = 98:2) could be used as a coupling partner with **1d** and **1e** in good yields (entries 12–13). Moreover, the coupling generally proceeds with an excellent *E*-selectivity when iodoalkenes are used. Total isomerization is observed for *Z*-starting iodoalkenes (entries 2, 4, 7, 8, 10). A similar tendency is observed for bromoalkenes, with the exception of 4-(2-bromovinyl)benzonitrile **3e** (*Z*/*E* = 98:2), which, intriguingly, did not lead to isomerization of the starting *Z* bond when coupled with nucleophile **1d**, entry 12, or to a slight isomerization when coupled with **1e**, entry 13. In all cases, efficient transference of both aryl groups from the starting *bis*-(aryl)manganese species has been observed.

## 3. Discussion

In order to rationalize some of the mechanistic features of the transformations reported in the first section, we focused our efforts on the coupling system involving various *bis*-(aryl)manganese nucleophiles (*bis*-mesitylmanganese and *bis*-phenylmanganese) with (2-bromovinyl)-trimethylsilane (**3b**). This choice has been motivated by the low cross-coupling yields obtained with this electrophile in the absence of the iron catalyst, which ascertains the requirement of an Fe-based catalysis for this coupling (see Table 2, entries 1, 3, 9 and 11).

**Figure 1:**
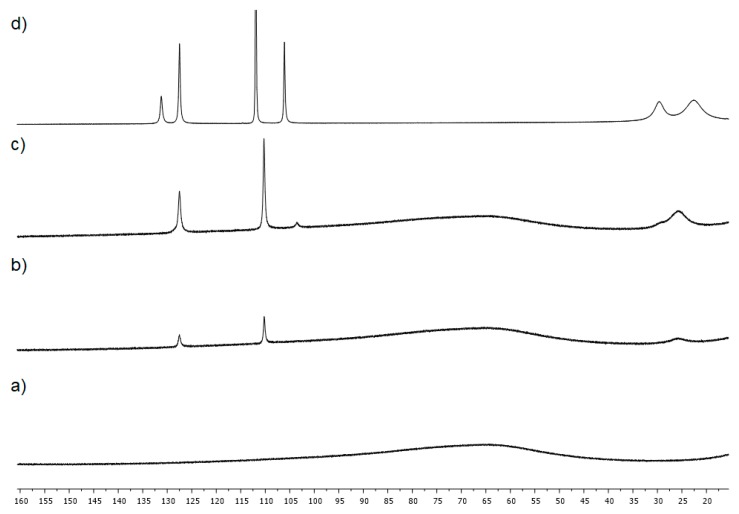
^1^H-NMR spectra (recorded at 25 °C in *d*_8_-THF) of a 0.08 M solution of (**a**) Mes_2_Mn, (**b**) Mes_2_Mn + 0.10 equiv. FeCl_2_, (**c**) Mes_2_Mn + 0.10 equiv. FeCl_2_ + 1.0 equiv. **3b**, (**d**) FeCl_2_ + 3.0 equiv. MesMgBr + 10 equiv. **3b**.

The *bis*-(mesityl)manganese reagent was prepared by adding MesMgBr (2.0 equiv.) into a solution of MnCl_2_•LiCl (1.0 equiv.) in THF at −5 °C within 1 h. ^1^H-NMR showed no free MesMgBr left. The spectra presented high signal-to-noise ratios and broad signals, due to the high paramagnetism of manganese(II) species, which could not be attributed to a specific molecule (Figure **1a**) [33]. High-spin organomanganese compounds are often reported to be NMR silent [34] or without NMR characterization at all [35,36,37]. Yet, after addition of a catalytic load of FeCl_2_ (0.10 equiv.) to the Mes_2_Mn solution, the *ate* complex [Mes_3_Fe^II^]^−^ was detected by ^1^H-NMR from the three signals at 127 ppm (s, 6H, *meta*-H of the Mes group), 110 ppm (s, 9H, *para*-CH_3_), and 26 ppm (bs, 18 H, *ortho*-CH_3_), as shown in Figure 1b. These signals attest to a strong paramagnetism, due to the high-spin (*S* = 2) configuration of this complex [38]. This proves a fast trans-metalation of the aryl groups from the manganese toward the iron(II) center. A similar result was obtained while adding an excess of the Mes_2_Mn solution onto Fe(acac)_3_ (0.10 equiv.) as an iron(III) precursor. [Mes_3_Fe^II^]^−^ was detected by ^1^H-NMR, showing that, when an iron(III) precursor is used, a first 1-electron reduction of the latter by the nucleophile can take place, affording an iron(II) species. This is in agreement with recent reports by Neidig [39] and by some of us [40] regarding the reduction of iron(III) salts by Grignard reagents as MeMgBr and PhMgBr. Accordingly, all the mechanistic studies discussed thereafter were performed using an iron(II) precursor.

Upon addition of the electrophile **3b** ((2-bromovinyl)trimethylsilane) to a mixture of FeCl_2_ and Mes_2_Mn at 25 °C, the signals corresponding to [Mes_3_Fe^II^]^−^ were observed to slowly decrease, affording [Mes_2_BrFe^II^]^−^, characterized by new signals at 128 ppm (s, 4H, *meta*-H of the Mes group), 104 ppm (s, 6H, *para*-CH_3_), and 29 ppm (bs, 12 H, *ortho*-CH_3_) (see Figure 1c) (this tricoordinate *ate* species also presents a high-spin *S* = 2 configuration) [38]. The same reaction was run at 25 °C for 1 h, then quenched, and analyzed by GC-MS, which proved formation of the desired cross-coupling product with a low conversion (ca. 20%). This is in fair agreement with the result given in Table 2, entry 11 (due to the high paramagnetism of the NMR-analyzed solution and due to the presence of non-deuterated solvents (THF solutions of organometallics), NMR monitoring of the coupling product formation could not be efficiently performed).

**Scheme 2:**
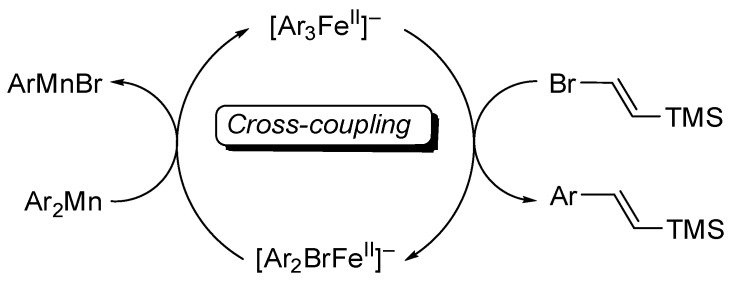
Proposed catalytic cycle for the aryl-alkenyl cross-coupling between Ar_2_Mn and an alkenyl bromide under Fe-catalytic conditions.

The same observations were made while performing the coupling of **3b** with MesMgBr as a sole nucleophilic partner in a Kumada-type reaction using FeCl_2_, as [Mes_3_Fe^II^]^−^ and [Mes_2_BrFe^II^]^−^ were also detected under these cross-coupling conditions (Figure 1d, in the absence of manganese, the signals of [Mes_2_BrFe^II^]^−^ shifted to 131, 106, and 30 ppm). These series of experiments, therefore, confirm that both [Ar_3_Fe^II^]^−^ and [Ar_2_BrFe^II^]^−^ *ate* complexes are part of a coupling catalytic cycle and [Ar_3_Fe^II^]^−^ can be involved in the activation step of the electrophile. The following catalytic cycle (Scheme 2) can be suggested, which echoes recent reports by Neidig on the Fe-catalyzed alkyl-alkenyl coupling reactions [39], and by ourselves on the benzyl-alkenyl coupling [26].

Thanks to the steric hindrance in the *ortho* positions, the *ate* [Mes_3_Fe^II^]^−^ species remains stable for hours at 25 °C [38,41]. Thus, the use of a mesityl nucleophile in the mechanistic experiments discussed earlier prevents any degradation of the Fe^II^ catalyst toward lower oxidation states. In order to delineate the influence of the formation of lower oxidation states on the system, additional investigations were, therefore, carried out using PhMgBr as a less hindered nucleophile [42].

**Figure 2:**
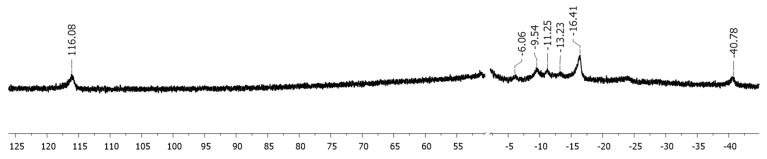
^1^H-NMR spectrum (recorded at −20 °C in *d*_8_-THF) of a 0.08 M solution of [Ph_3_Fe^II^]^−^ followed by addition of **3b** (10 equiv.). Spectrum recorded 20 min after addition of **3b**.

First, [Ph_3_Fe^II^]^−^ was generated at −20 °C by stoichiometric trans-metalation between FeCl_2_ and 3.0 equiv. of PhMgBr, and characterized by its ^1^H-NMR signals at 116 and −41 ppm. As we recently reported, [Ph_3_Fe^II^]^−^ is stable for more than 1 h at this temperature [40,41]. Its fate upon addition of an excess of the electrophile **3b** (10 equiv.) was then monitored by paramagnetic ^1^H-NMR. [Ph_3_Fe^II^]^−^ reacted rapidly, as attested by the decrease of its resonances (ca. 75% of the starting [Ph_3_Fe^II^]^−^ reacted after 10 min). The reaction was quenched after 1 h, and the GC-MS confirmed formation of the cross-coupling product, which confirms that [Ph_3_Fe^II^]^−^ was able to react with **3b** in a cross-coupling process, akin to [Mes_3_Fe^II^]^−^. Moreover, several transient resonances in the −15/−5 ppm area could also be detected in the course of the reaction (see Figure 2). These elusive resonances quickly disappeared, and were not detected after 30 min at −20 °C. These signals echo the formation of (η^2^-alkene)_n_-Fe^0^ intermediates, as recently reported by Deng, which exhibit similar resonances [43]. This suggests that Fe^0^ species are formed in situ by 2-electron reductive elimination from [Ph_3_Fe^II^]^−^, which is in agreement with a recent report by some of us demonstrating that the evolution of [Ph_3_Fe^II^]^−^ led to the formation of a distribution of Fe^0^ and Fe^I^ oxidation states (identified as (η^4^-arene)_2_Fe^0^ and [(η^6^-arene)Fe^I^(Ph)_2_]^−^, “arene” being an aromatic ligand present in the bulk medium (e.g., C_6_H_6_ or C_6_H_5_-C_6_H_5_ coming from the oxidation of PhMgBr). Fe^0^ being preponderantly formed [40,41]). Those Fe^0^ intermediates would then be trapped by alkene ligands present in the bulk medium, which leads to the observed resonances.

**Figure 3:**
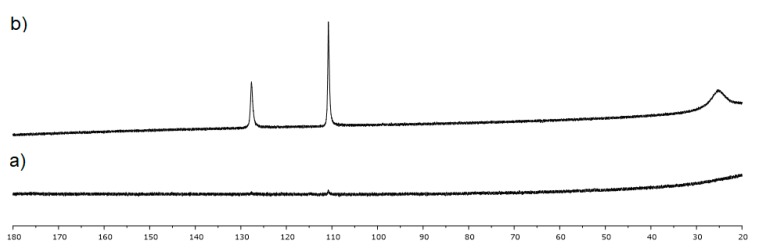
^1^H-NMR spectra (recorded at 25 °C in *d*_8_-THF) of a 0.08 M solution of (**a**) FeCl_2_ + 2.0 equiv. PhMgBr, + 1.0 equiv. MesMgBr after 10 min agitation at RT. (**b**) Same tube, + 3.0 equiv. **3b**, after 30 min.

Then, the reactivity of the low valent Fe^0^ and Fe^I^ oxidation states in the reaction medium was investigated. Following one of our recent procedures, reduction of Fe^II^ into a distribution of Fe^0^ and Fe^I^ species was performed, by fast trans-metalation between FeCl_2_ and PhMgBr (2.0 equiv.) at room temperature [40,41]. After 10 min, 1.0 equiv. of MesMgBr was added. The ^1^H-NMR spectrum showed no signal in the 100–150 ppm area, which attests to the absence of any Mes-Fe^II^ species, which shows that all the starting Fe^II^ was reduced by PhMgBr (Figure 3a).

The addition of 3.0 equiv. of the electrophile **3b** to the in situ generated solution of Fe^0^ and Fe^I^ species led to a color change of the sample, which turned from dark brown to yellow. The ^1^H-NMR spectrum showed that, after 30 min, ca. 20% of the iron contained in the solution was converted into [Mes_3_Fe^II^]^−^ (Figure 3b). The presence of **3b**, therefore, allows a re-oxidation of the reduced Fe^0^ and/or Fe^I^ species to the Fe^II^ oxidation state, the latter being trapped by trans-metalation with MesMgBr to afford [Mes_3_Fe^II^]^−^. The re-oxidation of low iron oxidation states by **3b** to the Fe^II^ stage was also confirmed by the observation of *bis*(trimethylsilyl)butadienes TMS-CH=CH-CH=CH-TMS (TMS-(CH)_4_-TMS, *E*/*E*; *Z*/*E*; *Z*/*Z*) in GC-MS, after catalytic reactions involving 10 mol% of FeCl_2_, PhMgBr, and 3b as coupling partners. Formation of TMS-(CH)_4_-TMS undoubtedly comes from the sacrificial monoelectronic reduction of the electrophile that permits re-oxidation of the low Fe^0^ and/or Fe^I^ oxidation states. TMS-(CH)_4_-TMS, moreover, also appears as a suitable ligand for Fe^0^ oxidation state, and a (η^4^-TMS-(CH)_4_-TMS)Fe^0^ complex might, thus, contribute to the group of high field resonances in the in the −15/−5 ppm area (Figure 2). Quantity of detected TMS-(CH)_4_-TMS represents ca. 10% of the quantity of a detected coupling product, which shows that this off-cycle sacrificial reduction pathway is not preponderant. By comparison, no traces of TMS-(CH)_4_-TMS were detected when using mesityl nucleophiles (conditions of Figure 1), attesting that no sacrificial 1-electron reduction of **3b** by oxidation states lower than Fe^II^ formed in situ occurred.

**Scheme 3:**
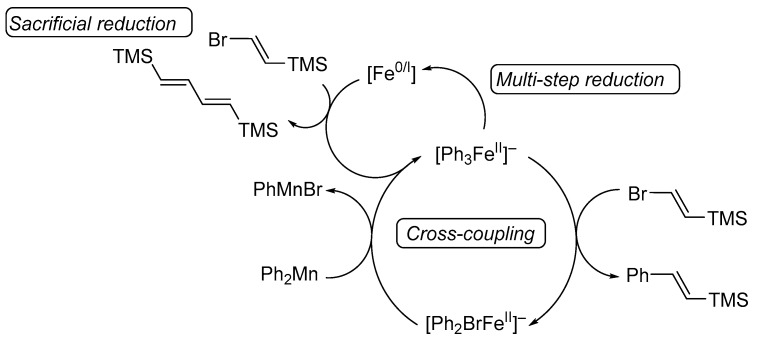
Proposed catalytic cycle and side reactions for the aryl-alkenyl cross-coupling between Ph_2_Mn and an alkenyl bromide under Fe-catalytic conditions.

Scheme 3 presents a summary of the competitive reactions that were observed in this work during the Fe-catalyzed coupling of Ph_2_Mn with (2-bromovinyl)-trimethylsilane (**3b**), taking into account the possibility of an off-cycle process involving in situ formed low iron oxidation states.

Kinetic studies will further be pursued in order to determine the global kinetics of the aryl-alkenyl cross-coupling reaction, and to examine the possibility for the low Fe^0^ and Fe^I^ oxidation states involved in a cross-coupling catalytic cycle, in addition to the off-cycle sacrificial reduction of the alkenyl electrophile evidenced herein. Such studies will also help analyze the mechanism of the activation of the C-X bond of the alkenyl halide. Isomerization toward the sterically more stable *E* coupling products may suggest the implication of an iron-based radical activation of the alkenyl halide, as observed in the case of alkyl electrophiles [44], albeit formation of the C_sp2_-centered radical is generally more energetically-demanding. Additionally, it cannot be excluded in an alternative scenario that isomerization of the C=C bond occurs after the coupling step, akin to the observations made by Jacobi von Wangelin for the iron-catalyzed isomerization of *Z*-olefins to their *E* analogues [45].

## 4. Materials and Methods

### 4.1. Materials and Instruments

All reactions, except otherwise noted, were carried out in flame-dried glassware equipped with magnetic stirring under an argon atmosphere using standard Schlenk techniques. To transfer solvents or reagents, syringes were used, which were purged three times with argon prior to use. After purification by flash column chromatography, products were concentrated using a rotary evaporator and, subsequently, dried under high vacuum. Indicated yields are isolated yields of compounds estimated to be >95% pure, as determined by ^1^H-NMR (25 °C) and capillary GC.

To examine the reaction progress of the performed reactions, GC-analysis of quenched hydrolyzed and iodolyzed reaction aliquots relative to an internal standard was used. For this purpose, small amounts of the reaction mixture were hydrolyzed using a saturated aqueous solution of NH_4_Cl, subsequently extracted with EtOAc, dried over MgSO_4_ and gaschromatographically quantified. To monitor the process of directed metalations and oxidative insertion reactions, small amounts of the reaction mixture were iodolyzed. A small quantity of iodine was dissolved in freshly distilled THF (0.50 mL), charged with the reaction mixture, and added to a solution of Na_2_S_2_O_3_. The mixture was extracted with EtOAc, dried over MgSO_4_, and was then gas chromatographically measured.

To determine the concentration of the different synthesized metallic reagents, iodometric titration was used. For this purpose, a known amount of iodine was charged with freshly distilled THF (1.00 mL) to give a deep red solution. The metallic reagent was added dropwise at 2 °C to the iodine solution until the red coloration went colorless. The concentration of the organometallic reagent could be calculated via the consumed volume of the reaction mixture and the amount of used iodine.

Thin layer chromatography (TLC) was implemented on alumina plates coated with SiO_2_ (Merck 60, F-254, Merck, Darmstadt, Germany). To visualize the spots of the different products, UV light was used.

Flash column chromatography was performed using SiO_2_ (0.04–0.06 mm, 230–400 mesh) from Merck.

^1^H-NMR, ^13^C-NMR, ^19^F-NMR, and 2D-NMR spectra were recorded on VARIAN Mercury 200, BRUKER ARX 300, VARIAN VXR 400 S, and BRUKER AMX 600 instruments (Bruker, Billerica, MA, USA). Chemical shifts are reported as δ-values in ppm relative to tetramethylsilane. The following abbreviations were used to characterize signal multiplicities: s (singlet), d (doublet), t (triplet), q (quartet), m (multiplet), and bs (broad singlet).

Mass spectroscopy: High resolution (HRMS) and low resolution (MS) spectra were recorded on a FINNIGAN MAT 95Q instrument (now Thermo Fisher company, Waltham, MA, USA). Electron impact ionization (EI) was conducted with an ionization energy of 70 eV. For coupled gas chromatography/mass spectrometry, a HEWLETT-PACKARD HP 6890 /MSD 5973 GC/MS system was used. Molecular fragments are reported starting at a relative intensity of 10%.

Infrared spectra (IR) were recorded from 4500 cm^−1^ to 650 cm^−1^ on a PERKIN ELMER Spectrum BX59343 instrument (Perkin Elmer, Wellesley, MA, USA). For detection, a SMITHS DETECTION DuraSamplIR IIDiamond ATR sensor (Smiths Detection, Hemel Hempstead, UK) was used. Wavenumbers are reported in cm^−1^ starting at an absorption of 10%.

Melting points (m.p.) were determined on a BÜCHI B-540 melting point apparatus (BÜCHI LabortechnikAG, Flawil, Switzerland) and are uncorrected. Compounds decomposing upon melting are indicated by (decomp.).

Gas chromatography was executed with machines of type Agilent Technologies 7890A GC-Systems with 6890 GC inlets, detectors (Agilent, Santa Clara, CA, USA), a GC oven, and a column of type HP 5 (Hewlett-Packard, 5% phenylmethylpolysiloxane; length: 10 m, diameter: 0.25 mm, film thickness: 0.2 µm).

Gas chromatography-Mass spectra were recorded on a networking system called Hewlett-Packard 6890/MSD 5973 GC/MS (Hewlett Packard, Palo Alto, CA, USA) with a column of type HP 5 (Hewlett-Packard, 5% phenylmethylpolysiloxane; length: 10m, diameter: 0.25 mm, film thickness: 0.2 µm).

### 4.2. Chemicals, Solvents, and Typical Procedures

All chemicals were purchased from commercial sources and were used without any further purification unless otherwise noted.

THF was continuously refluxed and freshly distilled from benzophenone ketyl under nitrogen. The freshly distilled THF was stored over a molecular sieve (4 Å) under argon. Solvents for column chromatography were distilled prior to use.

#### 4.2.1. Typical Procedure for the One-Pot Preparation of *Bis*-(aryl)manganese Reagents **1a**–**g**

A dry and argon-flushed Schlenk-tube, equipped with a magnetic stirring bar and a rubber septum, was charged with LiCl (0.610 g, 14.4 mmol, 2.4 equiv.), heated to 450 °C under high vacuum, and then cooled to room temperature. After being switched to argon, the same procedure was applied after MnCl_2_ was added (453 mg, 3.60 mmol, 0.6 equiv.). After cooling to room temperature, magnesium turnings were added (0.350 g, 14.4 mmol, 2.4 equiv.), which was followed by freshly distilled THF (12 mL). After the reaction mixture was cooled to −5 °C, the aryl bromides **2a**–**g** were then added dropwise using 1 mL syringes (6.0 mmol, 1.0 equiv., addition time: 1 min) and the reaction mixture was stirred until a complete conversion of the starting material was observed. The reaction progress was monitored by GC-analysis of hydrolyzed and iodolyzed aliquots.

When the metalation was completed, the concentration of the *bis*-(aryl)manganese species was determined by titration against iodine in freshly distilled THF. The black solutions of the aryl reagents **1a**–**g** were then separated from the magnesium turnings using a syringe and, subsequently, transferred into another pre-dried and argon-flushed Schlenk-tube, which was cooled to −5 °C. After a titration against iodine in freshly distilled THF was performed, the reagent was ready to use for Cross-Couplings.

#### 4.2.2. Typical Procedure for the Cross-Coupling Reactions of *Bis*-(aryl)manganese Reagents **1a**–**g** with Different Electrophiles **3a**–**e**

A pre-dried and argon-flushed Schlenk-tube equipped with a magnetic stirring bar and a rubber septum was charged with Fe(acac)_3_ (35 mg, 0.10 mmol, 10 mol%), the corresponding electrophile (**3a**–**e**, 1.0 mmol, 1.0 equiv.), tetradecane as internal standard (50 µL) and freshly distilled THF (1.0 mL) as solvent. The reaction mixture was cooled to 0 °C and the *bis-*(aryl)manganese solution (**1a**–**g**, 0.6 equiv.) was added dropwise whereupon a color change to dark brown could be recognized. After the addition was complete, the reaction mixture was stirred for a given time at room temperature and the completion of the cross-coupling reaction was monitored by GC-analysis of hydrolyzed aliquots. Thereupon, a saturated aqueous solution of NH_4_Cl was added and the aqueous layer was extracted with EtOAc (3 × 100 mL). The combined organic layers were dried over MgSO_4_, filtered, and concentrated under reduced pressure. Purification of the crude products by flash column chromatography afforded the desired cross-coupling reaction products (**4a**–**k**, **4m**, **4n**).

### 4.3. Studies on the Catalytically Active Species and Catalytic Cycle

All the samples were prepared in a recirculating JACOMEX inert atmosphere (Ar) drybox and vacuum Schlenk lines. Glassware was dried overnight at 120 °C before use. NMR spectra were obtained using a Bruker DPX 400 MHz spectrometer (Bruker, Billerica, MA, USA). Chemical shifts for ^1^H-NMR spectra were referenced to solvent impurities (herein, THF). NMR tubes were equipped with a J. Young valves were used for all ^1^H-NMR experiments and catalytic tests. The GC-MS analysis was performed using *n*-decane as an internal standard. The reaction media aliquots were quenched by the addition of distilled water under air. The organic products were extracted using DCM, and injected into the GC-MS. Mass spectra were recorded on a Hewlett-Packart HP 5973 mass spectrometer (Hewlett Packard, Palo Alto, CA, USA) via a GC-MS coupling with a Hewlett-Packart HP 6890 chromatograph (Hewlett Packard, Palo Alto, CA, USA) equipped with a capillary column HP-5MS (50 m × 0.25 mm × 0.25 µm, Hewlett Packard, Palo Alto, CA, USA). Ionisation was due to an electronic impact (EI, 70 eV).

## 5. Conclusions

In summary, various functionalized *bis*-(aryl)manganese species have been readily prepared in one-pot conditions from the corresponding aryl bromides by inserting magnesium in the presence of LiCl and in situ trans-metalation with MnCl_2_ in THF at −5 °C within 2 h. These *bis*-(aryl)manganese reagents have been allowed to undergo smooth iron-catalyzed cross-couplings using 10 mol% Fe(acac)_3_ and various functionalized alkenyl iodides and bromides at 25 °C for 1 h. Mechanistic investigations carried out by ^1^H-NMR showed that *ate*-iron(II) species [Ar_3_Fe^II^]^−^ are formed by trans-metalation of the *bis-*(aryl)manganese reagent with the iron catalyst, and that they can react with alkenyl bromides to afford the expected cross-coupling product. Low-valent Fe^0^ and Fe^I^ oxidation states can also be formed by the reduction of the *ate*-iron(II) catalyst under these conditions. This leads to the sacrificial reduction of the alkenyl electrophile via an off-cycle pathway, which partly regenerates the Fe^II^ oxidation state, where the latter is able to enter a new catalytic cycle.

## Figures and Tables

**Table 1:**
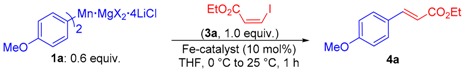
Catalyst screening of the reaction between the *bis*-(aryl)manganese reagent **1a** and (*Z*)-ethyl 3-iodoacrylate (**3a**).

Entry	Catalyst (10 mol%)	Yield (%) ^a^
1	none	60 ^b^
2	FeBr_2_	42
3	FeCl_3_	54
4	FeBr_3_	57
5	FeCl_2_	64
6	Fe(acac)_2_	67
7	Fe(acac)_3_ (>99% purity)	79

^a^ Yield of analytically pure product. ^b^ A *Z/E* = 50:50 mixture of **4a** was obtained.

**Table 2:**
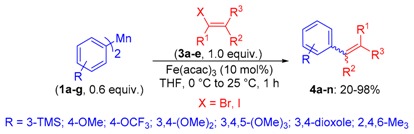
Iron-catalyzed couplings of *bis*-(aryl)manganese (**1a**–**g**) ^a^ with alkenyl electrophiles (**3a**–**e**).

Entry	Ar_2_Mn	Electrophile	Yield (%) ^b^	Entry	Ar_2_Mn	Electrophile	Yield (%) ^b^
1	**1a**	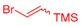 **3b**: *Z*/*E* = 10:90	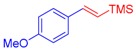 **4b**: 98, *Z*/*E* = 1:99 (24, *Z*/*E* = 20:80) ^c^	8	**1e**	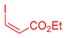 **3a**	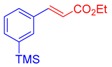 **4i**: 64, *Z*/*E* = 1:99 (51, *Z*/*E* = 57:43) ^c^
2	**1b**	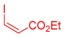 **3a**	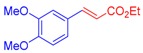 **4c**: 69, *Z*/*E* = 1:99 (58, *Z*/*E* = 69:31) ^c^	9	**1f**	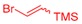 **3b**: *Z*/*E* = 10:90	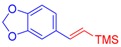 **4j**: 78, *Z*/*E* = 1:99 (39, *Z*/*E* = 20:80) ^c^
3	**1c**	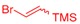 **3b**: *Z*/*E* = 10:90	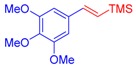 **4d**: 80, *Z*/*E* = 1:99 (8, *Z*/*E* = 1:99) ^c^	10	**1f**	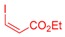 **3a**	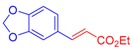 **4k**: 84, *Z*/*E* = 1:99 (48, *Z*/*E* = 99:1) ^c^
4	**1c**	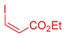 **3a**	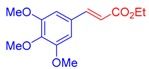 **4e**: 57, *Z*/*E* = 1:99 (66, *Z*/*E* = 72:28) ^c^	11	**1g**	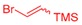 **3b**: *Z*/*E* = 10:90	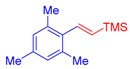 **4l**: ^d^ 20, *Z*/*E* = 21:79 91, *Z*/*E* = 5:95 ^e^ (traces, *Z*/*E* = 50:50) ^c^
5	**1c**	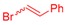 **3c**: *Z*/*E* = 18:82	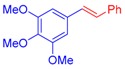 **4f**: 82, *Z*/*E* = 1:99 (80, *Z*/*E* = 53:47) ^c^	12	**1d**	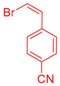 **3e**: *Z*/*E* = 98:2	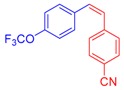 **4m**: 74, *Z*/*E* = 98:2 (19, *Z*/*E* = 73:27) ^c^
6	**1c**	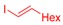 **3d**	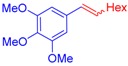 **4g**: 87, *Z*/*E* = 9:91 (77, *Z*/*E* = 4:96) ^c^	13	**1e**	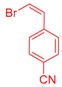 **3e**: *Z*/*E* = 98:2	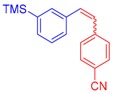 **4n**: 66, *Z*/*E* = 70:30 (41, *Z*/*E* = 72:28)^c^
7	**1d**	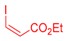 **3a**	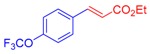 **4h**: 77, *Z*/*E* = 1:99 (74, *Z*/*E* = 81:19) ^c^				

^a^ For clarity reasons, the magnesium salt has been omitted. ^b^ Yield of analytically pure product. ^c^ In parentheses, yield and *Z/E* ratio obtained without catalysis. ^d^ Yields determined by GC and ^1^H-NMR. ^e^ After 18 h.

## Supplementary Materials

The following are available online. Additional synthesis and characterization (NMR, IR, HRMS, m.p.) data are available online.
