# Supplementary material for: Iron-Catalyzed Cross-Coupling of Bis-(aryl)manganese Nucleophiles with Alkenyl Halides: Optimization and Mechanistic Investigations
Source: Molecules. 2020 Feb 7;25(3):723. doi: 10.3390/molecules25030723 (PMC7037184; doi:10.3390/molecules25030723)

Iron-Catalyzed Cross-Coupling of *Bis*-(aryl)manganese Nucleophiles with Alkenyl Halides: Optimization and Mechanistic Investigations

Lidie Rousseau [1,2], Alexandre Desaintjean [3], Paul Knochel [3] and Guillaume Lefèvre *[1]

[1] Chimie ParisTech, PSL University, CNRS, Institute of Chemistry for Life and Health Sciences i-CLeHS FRE2027, CSB2D, 75005 Paris, France
[2] NIMBE, CEA, CNRS, Univ. Paris-Saclay, 91191 Gif-sur-Yvette, France
[3] Department of Chemistry, Ludwig-Maximilians-Universitat München, Butenandstr. 5-13, Haus F, 81377 Munich, Germany

* guillaume.lefevre@chimieparistech.psl.eu

Supporting Information

Table of Contents

General information 2
Typical Procedures 3
Additional Results 4
Preparation of *Bis*-(aryl)manganese Reagents (**1a-g**) 4
Fe-Catalyzed Cross-Coupling Reactions of *Bis*-(aryl)manganese Reagents **1a-g** with different Electrophiles (**3a-e**) 5
List of abbreviations 13
NMR spectra of compounds **4a-n** 14

General information

All reactions, except otherwise noted, were carried out in flame-dried glassware equipped with magnetic stirring under argon atmosphere using standard *Schlenk* techniques. To transfer solvents or reagents, syringes were used, which were purge three times with argon prior to use. After purification by flash column chromatography, products were concentrated using a rotary evaporator and subsequently dried under high vacuum. Indicated yields are isolated yields of compounds estimated to be > 95 % pure as determined by ^{1}H-NMR (25 °C) and capillary GC.

To examine the reaction progress of the performed reactions, GC-analysis of quenched hydrolyzed and iodolyzed reaction aliquots relative to an internal standard was used. For this purpose, small amounts of the reaction mixture were hydrolyzed using a saturated aqueous solution of NH_4Cl, subsequently extracted with EtOAc, dried over $MgSO_4$ and gaschromatographically quantified. To monitor the process of directed metalations and oxidative insertion reactions small amounts of the reaction mixture were iodolyzed. A small quantity of iodine was dissolved in freshly distilled THF (0.50 mL), charged with the reaction mixture and a solution of $Na_2S_2O_3$ was added. The mixture was extracted with EtOAc, dried over $MgSO_4$ and was then gaschromatographically measured.

To determine the concentration of the different synthesized metallic reagents iodometric titration was used. For this purpose, a known amount of iodine was charged with freshly distilled THF (1.00 mL) to give a deep red solution. The metallic reagent was added dropwise at 2 °C to the iodine solution until the red coloration went to colorless. The concentration of the organometallic reagent could be calculated *via* the consumed volume of the reaction mixture and the amount of used iodine.

Chromatography

Thin layer chromatography (TLC) was implemented on alumina plates coated with SiO_2 (Merck 60, F-254). To visualize the spots of the different products, UV light was used.

Flash column chromatography was performed using SiO_2 (0.04-0.06 mm, 230-400 mesh) from Merck.

Analytics

^{1}H NMR, ^{13}C NMR, ^{19}F NMR and 2D NMR spectra were recorded on VARIAN Mercury 200, BRUKER ARX 300, VARIAN VXR 400 S and BRUKER AMX 600 instruments. Chemical shifts are reported as δ-values in ppm relative to tetramethylsilane. The following abbreviations were used to characterize signal multiplicities: s (singlet), d (doublet), t (triplet), q (quartet), m (multiplet).

Mass spectroscopy: High resolution (HRMS) and low resolution (MS) spectra were recorded on a FINNIGAN MAT 95Q instrument. Electron impact ionization (EI) was conducted with an ionization energy of 70 eV. For coupled gas chromatography / mass spectrometry, a HEWLETT-PACKARD HP 6890 /MSD 5973 GC/MS system was used. Molecular fragments are reported starting at a relative intensity of 10%.

Infrared spectra (IR) were recorded from 4500 cm^{-1} to 650 cm^{-1} on a PERKIN ELMER Spectrum BX59343 instrument. For detection, a SMITHS DETECTION DuraSamplIR IIDiamond ATR sensor was used. Wavenumbers are reported in cm^{-1} starting at an absorption of 10%.

Melting points (m.p.) were determined on a BÜCHI B-540 melting point apparatus and are uncorrected. Compounds decomposing upon melting are indicated by (decomp.).

Gas chromatography was executed with machines of type Agilent Technologies 7890A GC-Systems with 6890 GC inlets, detectors, a GC oven and a column of type HP 5 (Hewlett-Packard, 5% phenylmethylpolysiloxane; length: 10m, diameter: 0.25 mm, film thickness: 0.2 µm).

Gas chromatography-Mass spectra were recorded on a networking system called Hewlett-Packard 6890/MSD 5973 GC/MS with a column of type HP 5 (Hewlett-Packard, 5% phenylmethylpolysiloxane; length: 10m, diameter: 0.25 mm, film thickness: 0.2 µm).

Chemicals

All chemicals were purchased from commercial sources and were used without any further purification unless otherwise noted.

Solvents

THF was continuously refluxed and freshly distilled from benzophenone ketyl under nitrogen. The freshly distilled THF was stored over molecular sieve (4Å) under argon. Solvents for column chromatography were distilled prior to use

Typical Reaction Procedures

Typical Procedure for the One-Pot Preparation of *Bis*-(aryl)manganese Reagents 1a-g (TP 1)

A dry and argon-flushed *Schlenk-tube*, equipped with a magnetic stirring bar and a rubber septum, was charged with LiCl (0.610 g, 14.4 mmol, 2.4 equiv), heated to 450 °C under high vacuum and then cooled to room temperature. After being switched to argon, the same procedure was applied after $MnCl_2$ was added (453 mg, 3.60 mmol, 0.6 equiv). After cooling to room temperature, magnesium turnings were added (0.350 g, 14.4 mmol, 2.4 equiv), followed by freshly distilled THF (12 mL). After the reaction mixture was cooled to -5 °C, the aryl bromides **2a-g** were then added drop by drop (6.0 mmol, 1.0 equiv) and the reaction mixture was stirred until a complete conversion of the starting material was observed. The reaction progress was monitored by GC-analysis of hydrolyzed and iodolyzed aliquots.
When the metalation was completed, the concentration of the *bis*-(aryl)manganese species was determined by titration against iodine in freshly distilled THF. The black solutions of the aryl reagents **1a-g** were then separated from the magnesium turnings using a syringe and subsequently transferred into another pre-dried and argon-flushed *Schlenk-tube*, which was cooled to -5 °C. After a titration against iodine in freshly distilled THF was performed, the reagent was ready to be used for Cross-Couplings.

Typical Procedure for the Cross-Coupling Reactions of *Bis*-(aryl)manganese Reagents 1a-g with different Elecrophiles 3a-e (TP 2)

A pre-dried and argon-flushed *Schlenk-tube* equipped with a magnetic stirring bar and a rubber septum was charged with $Fe(acac)_3$ (35 mg, 0.10 mmol, 10 mol%), the corresponding electrophile (**3a-e**, 1.00 mmol, 1.0 equiv), tetradecane as internal standard (50 µL) and freshly distilled THF (1.0 mL) as solvent. The reaction mixture was cooled to 0 °C and the *bis*-(aryl)manganese solution (**1a-g**, 0.6 equiv) was added dropwise whereupon a color change to dark brown could be recognized. After the addition was complete, the reaction mixture was stirred for a given time at room temperature and the completion of the cross-coupling reaction was monitored by GC-analysis of hydrolyzed aliquots. Thereupon, a saturated aqueous solution of NH_4Cl was added and the aqueous layer was extracted with EtOAc (3 x

100 mL). The combined organic layers were dried over $MgSO_4$, filtered and concentrated under reduced pressure. Purification of the crude products by flash column chromatography afforded the desired cross-coupling reaction products (**4a-n**). Product **4l** was not isolated and its yield was determined by GC and ^1H NMR.

Preparation of *Bis*-(aryl)manganese Reagents (1a-g)

Synthesis of *Bis*-(4-methoxyphenyl)manganese (1a)

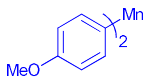

Based on **TP1**, LiCl (814 mg, 19.2 mmol, 2.40 equiv), magnesium turnings (467 mg, 19.2 mmol, 2.40 equiv), $MnCl_2$ (605 mg, 4.8 mmol, 0.60 equiv), THF (16.0 mL) and 1-bromo-4-methoxybenzene (**2a**, 1.496 g, 1.00 mL, 8.0 mmol, 1.00 equiv) were used. After stirring for 1 h at the given temperature, the concentration of *bis*-(4-methoxyphenyl)manganese (**1a**) was determined against iodine in THF (0.25 M, 87%).

Synthesis of *Bis*-(3,4-dimethoxyphenyl)manganese (1b)

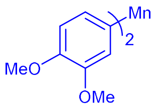

Based on **TP1**, LiCl (305 mg, 7.2 mmol, 2.40 equiv), magnesium turnings (175 mg, 7.2 mmol, 2.40 equiv), $MnCl_2$ (227 mg, 1.8 mmol, 0.60 equiv), THF (6.0 mL) and 5-bromo-1,2-dimethoxybenzene (**7i**, 868 mg, 3.0 mmol, 1.00 equiv) were used. After stirring for 2 h, the concentration of *bis*-(3,4-dimethoxyphenyl)manganese (**1b**) was determined against iodine in THF (0.15 M, 30%).

Synthesis of *Bis*-(3,4,5-trimethoxyphenyl)manganese (1c)

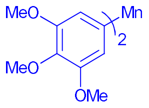

Based on **TP1**, LiCl (814 mg, 19.2 mmol, 2.40 equiv), magnesium turnings (467 mg, 19.2 mmol, 2.40 equiv), $MnCl_2$ (605 mg, 4.8 mmol, 0.60 equiv), THF (16.0 mL) and 5-bromo-1,2,3-trimethoxybenzene (**2c**, 1.977 g, 8.0 mmol, 1.00 equiv) were used. After stirring for 1 h at the given temperature, the concentration of *bis*-(3,4,5-trimethoxyphenyl)manganese (**1c**) was determined against iodine in THF (0.23 M, 68%).

Synthesis of *Bis*-(4-(trifluoromethoxy)phenyl)manganese (1d)

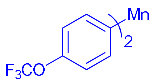

Based on **TP1**, LiCl (814 mg, 19.2 mmol, 2.40 equiv), magnesium turnings (467 mg, 19.2 mmol, 2.40 equiv), $MnCl_2$ (605 mg, 4.8 mmol, 0.60 equiv), THF (16.0 mL) and 1-bromo-4-(trifluoromethoxy)benzene

(**2d**, 1.928 g, 1.19 mL, 8.0 mmol, 1.00 equiv) were used. After stirring for 1 h at the given temperature, the concentration of *bis*-(4-(trifluoromethoxy)phenyl)manganese (**1d**) was determined against iodine in THF (0.22 M, 74%).

Synthesis of *Bis*-(3-(Trimethylsilyl)phenyl)manganese (1e)

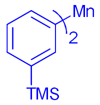

Based on **TP1**, LiCl (305 mg, 7.2 mmol, 2.40 equiv), magnesium turnings (175 mg, 7.2 mmol, 2.40 equiv), $MnCl_2$ (227 mg, 1.8 mmol, 0.60 equiv), THF (6.0 mL) and (3-bromophenyl)trimethylsilane (**2e**, 688 mg, 0.57 mL, 3.0 mmol, 1.00 equiv) were used. After stirring for 1 h at the given temperature, the concentration of *bis*-(3-(Trimethylsilyl)phenyl)manganese (**1e**) was determined against iodine in THF (0.13 M, 52%).

Synthesis of *Bis*-benzo[d][1,3]dioxol-5-ylmanganese (1f)

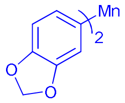

Based on **TP1**, LiCl (814 mg, 19.2 mmol, 2.40 equiv), magnesium turnings (467 mg, 19.2 mmol, 2.40 equiv), $MnCl_2$ (605 mg, 4.8 mmol, 0.60 equiv), THF (16.0 mL) and 5-bromobenzo[d][1,3]dioxole (**2f**, 1.608 g, 0.95 mL, 8.0 mmol, 1.00 equiv) were used. After stirring for 1 h at the given temperature, the concentration of *bis*-benzo[d][1,3]dioxol-5-ylmanganese (**1f**) was determined against iodine in THF (0.29 M, 79%).

Synthesis of *Bis*-(mesityl)manganese (1g)

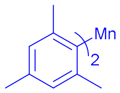

Based on **TP1**, LiCl (814 mg, 19.2 mmol, 2.40 equiv), magnesium turnings (467 mg, 19.2 mmol, 2.40 equiv), $MnCl_2$ (605 mg, 4.8 mmol, 0.60 equiv), THF (16.0 mL) and mesityl bromide (1.592 g, 1.22 mL, 8.0 mmol, 1.00 equiv) were used. After stirring for 1.5 h at the given temperature, the concentration of *bis*-(mesityl)manganese was determined against iodine in THF (0.27 M, 83%).

Fe-Catalyzed Cross-Coupling Reactions of *Bis*-(aryl)manganese Reagents 1a-g with different Electrophiles (3a-e)

Synthesis of (*E*)-Ethyl 3-(4-methoxyphenyl)acrylate (4a)

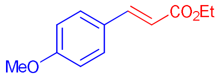

In relation to **TP2**, for this reaction Fe(acac)$_3$ (35 mg, 0.10 mmol, 10 mol%), (*Z*)-ethyl 3-iodoacrylate (**3a**, 226 mg, 0.13 mL, 1.00 mmol, 1.00 equiv) and freshly distilled THF (1.0 mL) were used. The reaction

mixture was cooled to 0 °C and *bis*-(4-methoxyphenyl)manganese (**1a**, 3.0 mL, 1.20 mmol, 1.20 equiv, 0.21 M) was added dropwise before the mixture was stirred for 1 h at the given temperature. Purification by flash column chromatography (SiO_2, *i*-hexane : EtOAc = 19:1, R_f = 0.20) afforded the desired cross-coupling reaction product **4a** (0.163 g, 0.79 mmol, 79%) as a pale-yellow solid.

m.p. : 50.5 °C.

^{1}H-NMR (400 MHz, $CDCl_3$, ppm) δ = 7.64 (d, *J* = 16.0 Hz, 1H), 7.47 (d, *J* = 8.7 Hz, 2H), 6.90 (d, *J* = 8.8 Hz, 2H), 6.30 (d, *J* = 16.0 Hz, 1H), 4.25 (q, *J* = 7.1 Hz, 2H), 3.83 (s, 3H), 1.33 (t, *J* = 7.1 Hz, 3H).

^{13}C-NMR (101 MHz, $CDCl_3$, ppm) δ = 167.4, 161.5, 144.4, 129.8, 127.3, 115.9, 114.4, 60.4, 55.5, 14.5.

IR (ATR, cm^{-1}) ṽ = 2972, 2930, 2898, 2870, 1708, 1632, 1604, 1512, 1458, 1442, 1372, 1316, 1302, 1288, 1252, 1206, 1164, 1096, 1026, 1006, 984, 930, 828, 778.

MS (EI, 70 eV, %) m/z = 206 (54), 178 (16), 162 (11), 161 (100), 134 (58), 133 (36), 118 (13), 89 (14).

HRMS (EI, 70 eV) m/z: calc. for **$C_{12}H_{14}O_3$**: 206.0943; found 206.0937.

Synthesis of (*E*)-(4-methoxystyryl)trimethylsilane (4b)

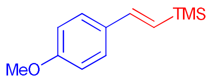

In relation to **TP2**, for this reaction Fe(acac)$_3$ (35 mg, 0.10 mmol, 10 mol%), 2-bromovinyltrimethylsilane (**3b**, *Z/E* = 10/90, 179 mg, 0.15 mL, 1.00 mmol, 1.00 equiv) and freshly distilled THF (1.0 mL) were used. The reaction mixture was cooled to 0 °C and *bis*-(4-methoxyphenyl)manganese (**1a**, 3.0 mL, 1.20 mmol, 1.20 equiv, 0.21 M) was added dropwise before the mixture was stirred for 0.5 h at the given temperature. Purification by flash column chromatography (SiO_2, *i*-hexane : EtOAc = 19:1, R_f = 0.71) afforded the desired cross-coupling reaction product **4b** (0.202 g, 0.98 mmol, 98%) as a pale-yellow solid.

m.p. : 51 °C.

^{1}H-NMR (400 MHz, $CDCl_3$, ppm) δ = 7.41 – 7.33 (m, 2H), 6.86 (m, 2H), 6.82 (d, *J* = 19.2 Hz, 1H), 6.31 (d, *J* = 19.1 Hz, 1H), 3.82 (s, 3H), 0.15 (s, 9H).

^{13}C-NMR (101 MHz, $CDCl_3$, ppm) δ = 159.7, 143.1, 131.5, 127.7, 126.8, 114.0, 55.5, -1.0.

IR (ATR, cm^{-1}) ṽ = 2954, 2898, 2836, 1606, 1572, 1508, 1488, 1464, 1440, 1418, 1304, 1296, 1244, 1196, 1172, 1106, 1032, 992, 864, 832, 796, 748, 738, 726, 690.

MS (EI, 70 eV, %) m/z = 209 (12), 206 (42), 193 (20), 192 (10), 191 (100), 183 (12), 176 (10), 175 (37), 165 (36).

HRMS (EI, 70 eV) m/z: calc. for **$C_{12}H_{18}OSi$**: 206.1127; found 206.1119.

Synthesis of (*E*)-Ethyl 3-(3,4-dimethoxyphenyl)acrylate (4c)

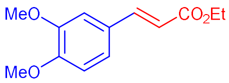

In relation to **TP2**, for this reaction Fe(acac)$_3$ (35 mg, 0.10 mmol, 10 mol%), (*Z*)-ethyl 3-iodoacrylate (**3a**, 226 mg, 0.13 mL, 1.00 mmol, 1.00 equiv) and freshly distilled THF (1.0 mL) were used. The reaction mixture was cooled to 0 °C and *bis*-(3,4-dimethoxyphenyl)manganese (**1b**, 4.0 mL, 0.60 mmol, 0.60 equiv, 0.15 M) was added dropwise before the mixture was stirred for 0.5 h at the given temperature. Purification by flash column chromatography (SiO_2, *i*-hexane : EtOAc = 8:2, R_f = 0.37) afforded the desired cross-coupling reaction product **4c** (0.165 g, 0.69 mmol, 69%) as a pale-yellow solid.

m.p. : 56.4 °C.

^{1}H-NMR (400 MHz, $CDCl_3$, ppm) δ = 7.61 (d, *J* = 15.9 Hz, 1H), 7.09 (dd, *J* = 8.3, 2.0 Hz, 1H), 7.04 (d, *J* = 2.0 Hz, 1H), 6.85 (d, *J* = 8.3 Hz, 1H), 6.30 (d, *J* = 15.9 Hz, 1H), 4.24 (q, *J* = 7.1 Hz, 2H), 3.90 (s, 6H), 1.32 (t, *J* = 7.1 Hz, 3H).

^{13}C-NMR (101 MHz, $CDCl_3$, ppm) δ = 167.3, 151.2, 149.3, 144.6, 127.6, 122.7, 116.1, 111.1, 109.7, 60.5, 56.0 (2 C), 14.5.

IR (ATR, cm^{-1}) ṽ = 2984, 2960, 2938, 2836, 1692, 1628, 1598, 1582, 1512, 1466, 1454, 1438, 1424, 1364, 1246, 1224, 1176, 1160, 1142, 1096, 1044, 1024, 986, 976, 968, 936, 870, 860, 802, 776, 752.

MS (EI, 70 eV, %) m/z = 237 (14), 236 (100), 191 (67), 164 (20), 105 (15), 70 (10), 61 (15), 44 (10), 43 (16), 42 (60).

HRMS (EI, 70 eV) m/z: calc. for **$C_{13}H_{16}O_4$**: 236.1049; found 236.1042.

Synthesis of (*E*)-Trimethyl(3,4,5-trimethoxystyryl)silane (4d)

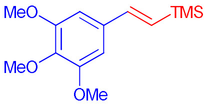

In relation to **TP2**, for this reaction Fe(acac)$_3$ (35 mg, 0.10 mmol, 10 mol%), 2-bromovinyltrimethylsilane (**3b**, *Z/E* = 10/90, 179 mg, 0.15 mL, 1.00 mmol, 1.00 equiv) and freshly distilled THF (1.0 mL) were used. The reaction mixture was cooled to 0 °C and *bis*-(3,4,5-trimethoxyphenyl)manganese (**1c**, 3.2 mL, 0.60 mmol, 0.60 equiv, 0.22 M) was added dropwise before the mixture was stirred for 0.5 h at the given temperature. Purification by flash column chromatography (SiO_2, *i*-hexane : EtOAc = 19:1, R_f = 0.29) afforded the desired cross-coupling reaction product **4d** (0.215 g, 0.80 mmol, 80%) as a white solid.

m.p. : 45 °C.

^{1}H-NMR (400 MHz, $CDCl_3$, ppm) δ = 6.79 (d, *J* = 19.0 Hz, 1H), 6.67 (s, 2H), 6.37 (d, *J* = 19.0 Hz, 1H), 3.87 (d, *J* = 17.1 Hz, 9H), 0.16 (s, 9H).

^{13}C-NMR (101 MHz, $CDCl_3$, ppm) δ = 153.4, 143.5, 138.3, 134.2, 128.9, 103.5, 61.0, 56.2, -1.1.

IR (ATR, cm^{-1}) ṽ = 3000, 2952, 2900, 2838, 2828, 1574, 1504, 1464, 1450, 1432, 1414, 1328, 1238, 1202, 1184, 1148, 1124, 1006, 996, 982, 862, 834, 804, 782, 742, 732, 690.

MS (EI, 70 eV, %) m/z = 267 (13), 266 (100), 251 (64), 239 (10), 236 (41), 235 (12), 221 (45), 220 (15), 219 (12), 205 (21), 194 (17), 179 (13), 161 (16), 89 (10).

HRMS (EI, 70 eV) m/z: calc. for $\mathbf{C_{14}H_{22}O_3Si}$: 266.1338; found 266.1326.

Synthesis of (*E*)-Ethyl 3-(3,4,5-trimethoxyphenyl)acrylate (4e)

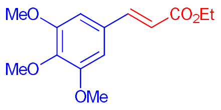

In relation to **TP2**, for this reaction Fe(acac)$_3$ (35 mg, 0.10 mmol, 10 mol%), (*Z*)-ethyl 3-iodoacrylate (**3a**, 226 mg, 0.13 mL, 1.00 mmol, 1.00 equiv) and freshly distilled THF (1.0 mL) were used. The reaction mixture was cooled to 0 °C and *bis*-(3,4,5-trimethoxyphenyl)manganese (**1c**, 3.2 mL, 0.60 mmol, 0.60 equiv, 0.22 M) was added dropwise before the mixture was stirred for 0.5 h at the given temperature. Purification by flash column chromatography (SiO_2, *i*-hexane : EtOAc = 19:1, R_f = 0.41) afforded the desired cross-coupling reaction product **4e** (0.151 g, 0.57 mmol, 57%) as a yellow-orange solid.

m.p. : 71.2 °C.

^{1}H-NMR (400 MHz, CDCl$_3$, ppm) δ = 7.59 (d, *J* = 15.9 Hz, 1H), 6.74 (s, 2H), 6.34 (d, *J* = 15.9 Hz, 1H), 4.25 (q, *J* = 7.1 Hz, 2H), 3.97 – 3.76 (m, 9H), 1.33 (t, *J* = 7.1 Hz, 3H).

^{13}C-NMR (101 MHz, CDCl$_3$, ppm) δ = 166.9, 153.4, 144.5, 140.1, 130.0, 117.5, 105.2, 61.0, 60.5, 56.1, 14.3.

IR (ATR, cm^{-1}) ṽ = 2974, 2944, 2928, 2838, 1700, 1632, 1582, 1504, 1470, 1452, 1432, 1414, 1340, 1310, 1272, 1242, 1174, 1148, 1118, 1034, 996, 982, 922, 872, 824, 790, 760.

MS (EI, 70 eV, %) m/z = 267 (14), 266 (100), 251 (55), 223 (15), 221 (19), 179 (11), 177 (21), 163 (21), 135 (10).

HRMS (EI, 70 eV) m/z: calc. for $\mathbf{C_{14}H_{18}O_5}$: 266.1154; found 266.1142.

Synthesis of (*E*)-1,2,3-trimethoxy-5-styrylbenzene (4f)

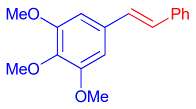

In relation to **TP2**, for this reaction Fe(acac)$_3$ (35 mg, 0.10 mmol, 10 mol%), 2-bromovinylbenzene (**3c**, *Z/E* = 18:82, 183 mg, 0.13 mL, 1.00 mmol, 1.00 equiv) and freshly distilled THF (1.0 mL) were used. The reaction mixture was cooled to 0 °C and *bis*-(3,4,5-trimethoxyphenyl)manganese (**1c**, 3.2 mL, 0.60 mmol, 0.60 equiv, 0.22 M) was added dropwise before the mixture was stirred for 0.5 h at the given temperature. Purification by flash column chromatography (SiO_2, *i*-hexane : EtOAc = 19:1, R_f = 0.12) afforded the desired cross-coupling reaction product **4f** (0.223 g, 0.82 mmol, 82%) as a pale-yellow solid.

m.p. : 106.6 °C.

^{1}**H-NMR (400 MHz, CDCl$_3$, ppm)** δ = 7.54 (d, *J* = 7.2 Hz, 2H), 7.39 (t, *J* = 7.6 Hz, 2H), 7.29 (tt, *J* = 6.8, 1.2 Hz, 1H), 7.18 – 6.95 (m, 2H), 6.77 (s, 2H), 3.95 (s, 6H), 3.91 (s, 3H).

^{13}**C-NMR (101 MHz, CDCl$_3$, ppm)** δ = 153.8, 138.4, 137.6, 133.5, 129.1, 129.0, 128.6, 128.0, 126.9, 104.1, 61.4, 56.6.

IR (ATR, cm^{-1}) ṽ = 2924, 2854, 1582, 1506, 1460, 1448, 1428, 1418, 1346, 1326, 1260, 1238, 1150, 1128, 1074, 1006, 984, 972, 846, 816, 784, 748, 692.

MS (EI, 70 eV, %) m/z = 271 (18), 270 (100), 256 (14), 255 (83), 195 (25), 181 (10), 180 (13), 167 (26), 165 (26), 153 (10), 152 (28), 141 (22), 115 (16).

HRMS (EI, 70 eV) m/z: calc. for **$C_{17}H_{18}O_3$**: 270.1256; found 270.1251.

Synthesis of 1,2,3-Trimethoxy-5-(oct-1-en-1-yl)benzene (4g)

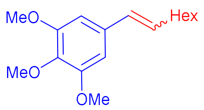

In relation to **TP2**, for this reaction Fe(acac)$_3$ (35 mg, 0.10 mmol, 10 mol%), (*E*)-1-iodo-1-octene (**3d**, 238 mg, 0.17 mL, 1.00 mmol, 1.00 equiv) and freshly distilled THF (1.0 mL) were used. The reaction mixture was cooled to 0 °C and *bis*-(3,4,5-trimethoxyphenyl)manganese (**1c**, 3.2 mL, 0.60 mmol, 0.60 equiv, 0.22 M) was added dropwise before the mixture was stirred for 1 h at the given temperature. Purification by flash column chromatography (SiO$_2$, *i*-hexane : EtOAc = 9:1, R_f = 0.36) afforded the desired cross-coupling reaction product **4g** (0.242 g, Z/E = 9/91, 0.87 mmol, 87%) as a pale-yellow oil.

^{1}**H-NMR (400 MHz, CDCl$_3$, ppm)** δ = 6.57 (s, 2H), 6.30 (d, *J* = 15.7 Hz, 1H), 6.14 (dt, *J* = 15.7, 6.8 Hz, 1H), 3.85 (d, *J* = 15.5 Hz, 9H), 2.26 – 2.10 (m, 2H), 1.71 – 1.19 (m, 8H), 0.89 (t, *J* = 6.9 Hz, 3H).

^{13}**C-NMR (101 MHz, CDCl$_3$, ppm)** δ = 153.4, 137.4, 133.9, 131.0, 129.7, 103.1, 61.1, 56.2, 33.1, 31.9, 29.5, 28.9, 22.8, 14.3.

IR (ATR, cm^{-1}) ṽ = 2954, 2926, 2872, 2854, 1580, 1506, 1454, 1430, 1416, 1340, 1324, 1236, 1184, 1152, 1124, 1104, 1042, 1008, 960, 844, 808, 780, 688.

MS (EI, 70 eV, %) m/z = 281 (13), 279 (14), 278 (79), 263 (12), 225 (21), 207 (54), 196 (11), 195 (11), 182 (23), 181 (53), 179 (33), 177 (16), 176 (100), 175 (14), 167 (13), 161 (32), 153 (18), 151 (30), 133 (12), 115 (15), 91 (27), 79 (15), 77 (11), 67 (12).

HRMS (EI, 70 eV) m/z: calc. for **$C_{17}H_{26}O_3$**: 278.1882; found 278.1871.

Synthesis of (*E*)-Ethyl 3-(4-(trifluoromethoxy)phenyl)acrylate (4h)

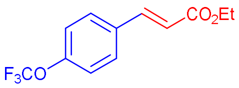

In relation to **TP2**, for this reaction Fe(acac)$_3$ (35 mg, 0.10 mmol, 10 mol%), (*Z*)-ethyl 3-iodoacrylate (**3a**, 226 mg, 0.13 mL, 1.00 mmol, 1.00 equiv) and freshly distilled THF (1.0 mL) were used. The reaction mixture was cooled to 0 °C and *bis*-(4-(trifluoromethoxy)phenyl)manganese (**1d**, 4.4 mL, 0.60 mmol,

0.60 equiv, 0.14 M) was added dropwise before the mixture was stirred for 0.5 h at the given temperature. Purification by flash column chromatography (SiO_2, *i*-hexane : EtOAc = 9:1, R_f = 0.50) afforded the desired cross-coupling reaction product **4h** (0.200 g, 0.77 mmol, 77%) as a colorless oil.

^{1}H-NMR (400 MHz, $CDCl_3$, ppm) δ = 7.65 (d, *J* = 16.0 Hz, 1H), 7.59 – 7.48 (m, 2H), 7.22 (d, *J* = 8.0 Hz, 2H), 6.41 (d, *J* = 16.0 Hz, 1H), 4.27 (q, *J* = 7.1 Hz, 2H), 1.34 (t, *J* = 7.1 Hz, 3H).

^{13}C-NMR (101 MHz, $CDCl_3$, ppm) δ = 166.8, 150.5 (q, *J* = 1.8 Hz), 142.9, 133.2, 129.6, 121.3, 120.5 (q, *J* = 258.0 Hz), 119.4, 60.8, 14.4.

^{19}F NMR (375 MHz, $CDCl_3$, ppm) δ = -57.8.

IR (ATR, cm^{-1}) $\tilde{v}$ = 2986, 1710, 1642, 1606, 1588, 1508, 1418, 1368, 1312, 1248, 1210, 1154, 1106, 1036, 1018, 980, 946, 922, 884, 836, 810, 798, 700, 668.

MS (EI, 70 eV, %) m/z = 260 (37), 232 (16), 215 (11), 214 (100), 187 (13), 186 (28), 101 (14).

HRMS (EI, 70 eV) m/z: calc. for **$C_{12}H_{11}F_3O_3$**: 260.0660; found 260.0646.

Synthesis of (*E*)-Ethyl 3-(3-(trimethylsilyl)phenyl)acrylate (4i)

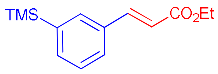

In relation to **TP2**, for this reaction Fe(acac)$_3$ (35 mg, 0.10 mmol, 10 mol%), (*Z*)-ethyl 3-iodoacrylate (**3a**, 226 mg, 0.13 mL, 1.00 mmol, 1.00 equiv) and freshly distilled THF (1.0 mL) were used. The reaction mixture was cooled to 0 °C and *bis*-(3-trimethylsilylphenyl)manganese (**1e**, 4.6 mL, 0.60 mmol, 0.60 equiv, 0.13 M) was added dropwise before the mixture was stirred for 1 h at the given temperature. Purification by flash column chromatography (SiO_2, *i*-hexane : EtOAc = 19:1, R_f = 0.44) afforded the desired cross-coupling reaction product **4i** (0.160 g, 0.64 mmol, 64%) as a pale-yellow oil.

^{1}H-NMR (400 MHz, $CDCl_3$, ppm) δ = 7.71 (d, *J* = 16.0 Hz, 1H), 7.66 (s, 1H), 7.56 – 7.49 (m, 2H), 7.37 (t, *J* = 7.5 Hz, 1H), 6.46 (d, *J* = 16.0 Hz, 1H), 4.28 (q, *J* = 7.1 Hz, 2H), 1.35 (t, *J* = 7.1 Hz, 3H), 0.29 (s, 9H).

^{13}C-NMR (101 MHz, $CDCl_3$, ppm) δ = 167.2, 145.1, 141.6, 135.4, 133.8, 133.3, 128.4, 128.4, 118.3, 60.6, 14.5, -1.1.

IR (ATR, cm^{-1}) $\tilde{v}$ = 2978, 2956, 1710, 1638, 1474, 1396, 1366, 1306, 1264, 1248, 1206, 1166, 1126, 1112, 1096, 1036, 982, 912, 900, 862, 834, 794, 752, 726, 684.

MS (EI, 70 eV, %) m/z = 251 (34), 248 (25), 234 (11), 233 (100), 205 (43), 187 (40), 131 (53), 115 (11), 75 (29), 73 (13).

HRMS (EI, 70 eV) m/z: calc. for **$C_{14}H_{20}O_2Si$**: 248.1233; found 248.1223.

Synthesis of (*E*)-(2-(benzo[d][1,3]dioxol-5-yl)vinyl)trimethylsilane (4j)

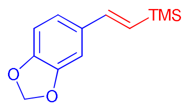

In relation to **TP2**, for this reaction Fe(acac)$_3$ (35 mg, 0.10 mmol, 10 mol%), 2-bromovinyltrimethylsilane (**3b**, *Z/E* = 10:90, 179 mg, 0.15 mL, 1.00 mmol, 1.00 equiv) and freshly distilled THF (1.0 mL) were used. The reaction mixture was cooled to 0 °C and *bis*-benzo[d][1,3]dioxol-5-ylmanganese (**1f**, 2.5 mL, 0.60 mmol, 0.60 equiv, 0.28 M) was added dropwise before the mixture was stirred for 0.5 h at the given temperature. Purification by flash column chromatography (SiO$_2$, *i*-hexane : EtOAc = 19:1, R$_f$ = 0.74) afforded the desired cross-coupling reaction product **4j** (0.171 g, 0.78 mmol, 78%) as a pale-yellow oil.

^{1}H-NMR (400 MHz, CDCl$_3$, ppm) δ = 7.00 (d, *J* = 1.7 Hz, 1H), 6.87 (dd, *J* = 8.0, 1.6 Hz, 1H), 6.78 (d, *J* = 18.9 Hz, 1H), 6.77 (s, 1H), 6.27 (d, *J* = 19.1 Hz, 1H), 5.95 (s, 2H), 0.15 (s, 9H).

^{13}C-NMR (101 MHz, CDCl$_3$, ppm) δ = 148.2, 147.6, 143.1, 133.3, 127.4, 121.5, 108.3, 105.6, 101.2, -1.0.

IR (ATR, cm^{-1}) ṽ = 2954, 2894, 1594, 1502, 1488, 1444, 1354, 1244, 1204, 1186, 1120, 1096, 1038, 982, 942, 928, 862, 834, 786, 758, 742, 728, 690.

MS (EI, 70 eV, %) m/z = 220 (67), 206 (10), 205 (100), 189 (20), 179 (10), 175 (63), 165 (10), 149 (17), 148 (10), 147 (26), 145 (17).

HRMS (EI, 70 eV) m/z: calc. for **$C_{12}H_{16}O_2Si$**: 220.0920; found 220.0911.

Synthesis of (*E*)-Ethyl 3-(benzo[d][1,3]dioxol-5-yl)acrylate (4k)

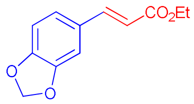

In relation to **TP2**, for this reaction Fe(acac)$_3$ (35 mg, 0.10 mmol, 10 mol%), (*Z*)-ethyl 3-iodoacrylate (**3a**, 226 mg, 0.13 mL, 1.00 mmol, 1.00 equiv) and freshly distilled THF (1.0 mL) were used. The reaction mixture was cooled to 0 °C and *bis*-benzo[d][1,3]dioxol-5-ylmanganese (**1f**, 2.5 mL, 0.60 mmol, 0.60 equiv, 0.28 M) was added dropwise before the mixture was stirred for 1 h at the given temperature. Purification by flash column chromatography (SiO$_2$, *i*-hexane : EtOAc = 19:1, R$_f$ = 0.25) afforded the desired cross-coupling reaction product **4k** (0.186 g, 0.84 mmol, 84%) as a white solid.

m.p. : 68.8 °C.

^{1}H-NMR (400 MHz, CDCl$_3$, ppm) δ = 7.58 (d, *J* = 15.9 Hz, 1H), 6.95 (ddd, *J* = 78.4, 42.1, 4.8 Hz, 3H), 6.25 (d, *J* = 15.9 Hz, 1H), 6.00 (s, 2H), 4.24 (q, *J* = 7.1 Hz, 2H), 1.32 (t, *J* = 7.1 Hz, 3H).

^{13}C-NMR (101 MHz, CDCl$_3$, ppm) δ = 167.3, 149.7, 148.5, 144.4, 129.0, 124.5, 116.4, 108.7, 106.6, 101.7, 60.5, 14.5.

IR (ATR, cm^{-1}) ṽ = 2922, 2854, 1702, 1640, 1610, 1504, 1490, 1474, 1448, 1440, 1368, 1356, 1242, 1192, 1174, 1142, 1118, 1096, 1028, 1002, 926, 854, 804.

MS (EI, 70 eV, %) m/z = 221 (13), 220 (100), 192 (23), 191 (18), 175 (80), 174 (12), 173 (11), 148 (64), 147 (23), 146 (18), 145 (82), 117 (33), 89 (45), 63 (10).

HRMS (EI, 70 eV) m/z: calc. for $\mathbf{C_{12}H_{12}O_4}$: 220.0736; found 220.0728.

Synthesis of (*Z*)-4-(4-(trifluoromethoxy)styryl)benzonitrile (4m)

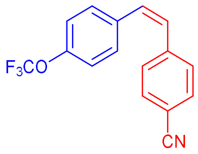

In relation to **TP2**, for this reaction Fe(acac)$_3$ (35 mg, 0.10 mmol, 10 mol%), 4-(2-bromovinyl)benzonitrile (**3e**, *Z/E* = 98:2, 208 mg, 1.00 mmol, 1.00 equiv) and freshly distilled THF (1.0 mL) were used. The reaction mixture was cooled to 0 °C and *bis*-(4-(trifluoromethoxy)phenyl)manganese (**1d**, 4.4 mL, 0.60 mmol, 0.60 equiv, 0.14 M) was added dropwise before the mixture was stirred for 1 h at the given temperature. Purification by flash column chromatography (SiO$_2$, *i*-hexane : EtOAc = 99:1, R$_f$ = 0.18) afforded the desired cross-coupling reaction product **4m** (0.214 g, 0.74 mmol, 74%) as a pale-yellow oil.

^{1}H-NMR (400 MHz, CDCl$_3$, ppm) δ = 7.58 – 7.45 (m, 2H), 7.37 – 7.28 (m, 2H), 7.24 – 7.15 (m, 2H), 7.13 – 7.03 (m, 2H), 6.72 (d, *J* = 12.2 Hz, 1H), 6.62 (d, *J* = 12.2 Hz, 1H).

^{13}C-NMR (101 MHz, CDCl$_3$, ppm) δ = 148.7, 141.7, 134.9, 132.3, 131.8, 130.4, 129.6, 129.4, 121.0, 120.5 (d, *J* = 257.5 Hz), 118.9, 111.0.

^{19}F NMR (375 MHz, CDCl$_3$, ppm) δ = -57.8.

IR (ATR, cm^{-1}) $\tilde{\nu}$ = 2228, 1605, 1507, 1407, 1252, 1210, 1198, 1156, 1110, 1018, 948, 922, 885, 832, 813, 779, 760, 741, 670.

MS (EI, 70 eV, %) m/z = 290 (17), 289 (98), 249 (11), 205 (17), 204 (100), 203 (32), 202 (36), 190 (31), 177 (21), 165 (16).

HRMS (EI, 70 eV) m/z: calc. for $\mathbf{C_{16}H_{10}F_3NO}$: 289.0714; found 289.0708.

Synthesis of 4-(3-(trimethylsilyl)styryl)benzonitrile (4n)

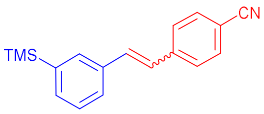

In relation to **TP2**, for this reaction Fe(acac)$_3$ (35 mg, 0.10 mmol, 10 mol%), 4-(2-bromovinyl)benzonitrile (**3e**, *Z/E* = 98:2, 208 mg, 1.00 mmol, 1.00 equiv) and freshly distilled THF (1.0 mL) were used. The reaction mixture was cooled to 0 °C and *bis*-(3-trimethylsilylphenyl)manganese (**1e**, 4.6 mL, 0.60 mmol, 0.60 equiv, 0.13 M) was added dropwise before the mixture was stirred for 1 h at the given temperature. Purification by flash column chromatography (SiO$_2$, *i*-hexane : EtOAc = 99:1, R$_f$ = 0.30) afforded the desired cross-coupling reaction product **4n** (0.182 g, *Z/E* = 7:3, 0.66 mmol, 66%) as a pale-yellow oil.

^{1}H-NMR (400 MHz, CDCl$_3$, ppm) δ = 7.67 – 7.46 (m, 3.3H), 7.41 – 7.31 (m, 3H), 7.28 – 7.16 (m, 2H), 7.10 (d, *J* = 16.4 Hz, 0.3H), 6.67 (ddd, *J* = 74.6, 12.2 Hz, 1.4H).

^{13}C-NMR (101 MHz, CDCl$_3$, ppm) δ = 142.5, 142.1, 141.4, 140.8, 135.6, 135.4, 134.0, 133.8, 133.7, 132.9, 132.8, 132.6, 132.3, 132.1, 129.7, 129.4, 128.4, 128.4, 128.0, 127.1, 127.0, 126.7, 119.2, 119.1, 110.6, 110.5, -1.0, -1.2.

IR (ATR, cm^{-1}) ṽ = 2954, 2226, 1603, 1504, 1414, 1403, 1377, 1261, 1248, 1176, 1112, 965, 926, 898, 884, 858, 831, 801, 750, 712, 689.

MS (EI, 70 eV, %) m/z = 280 (27), 277 (40), 263 (17), 262 (100).

HRMS (EI, 70 eV) m/z: calc. for **$C_{18}H_{19}NSi$**: 277.1287; found 277.1280.

List of Abbreviations

acac	acetylacetonate
d	doublet
equiv	equivalent
Et	ethyl
GC	gas chromatography
h	hour
hept	heptuplet
Hex	hexyl
HRMS	high resolution mass spectrometry
Hz	hertz
IR	infrared spectra
m	multiplet
min	minute
M.p.	melting point
MS	mass spectrometry
NMR	nuclear magnetic resonance
OMe	methoxy
ppm	parts per million
q	quartet
R_f	retention factor
s	singulet
t	triplet
THF	tetrahydrofuran
TLC	thin layer chromatography
TMS	trimethylsilyl
TP	typical procedure
UV	ultraviolet lig

NMR spectra of compounds 4a-n

(*E*)-Ethyl 3-(4-methoxyphenyl)acrylate (4a)

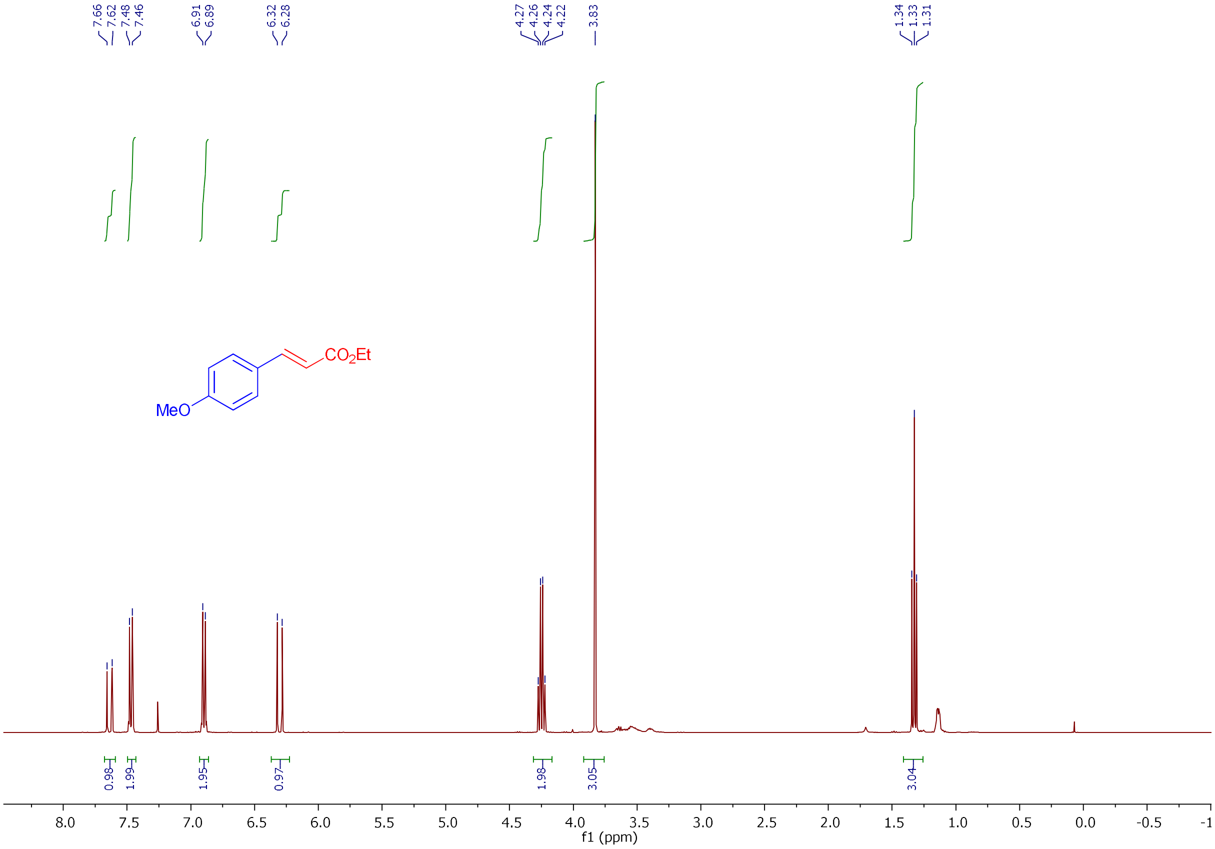

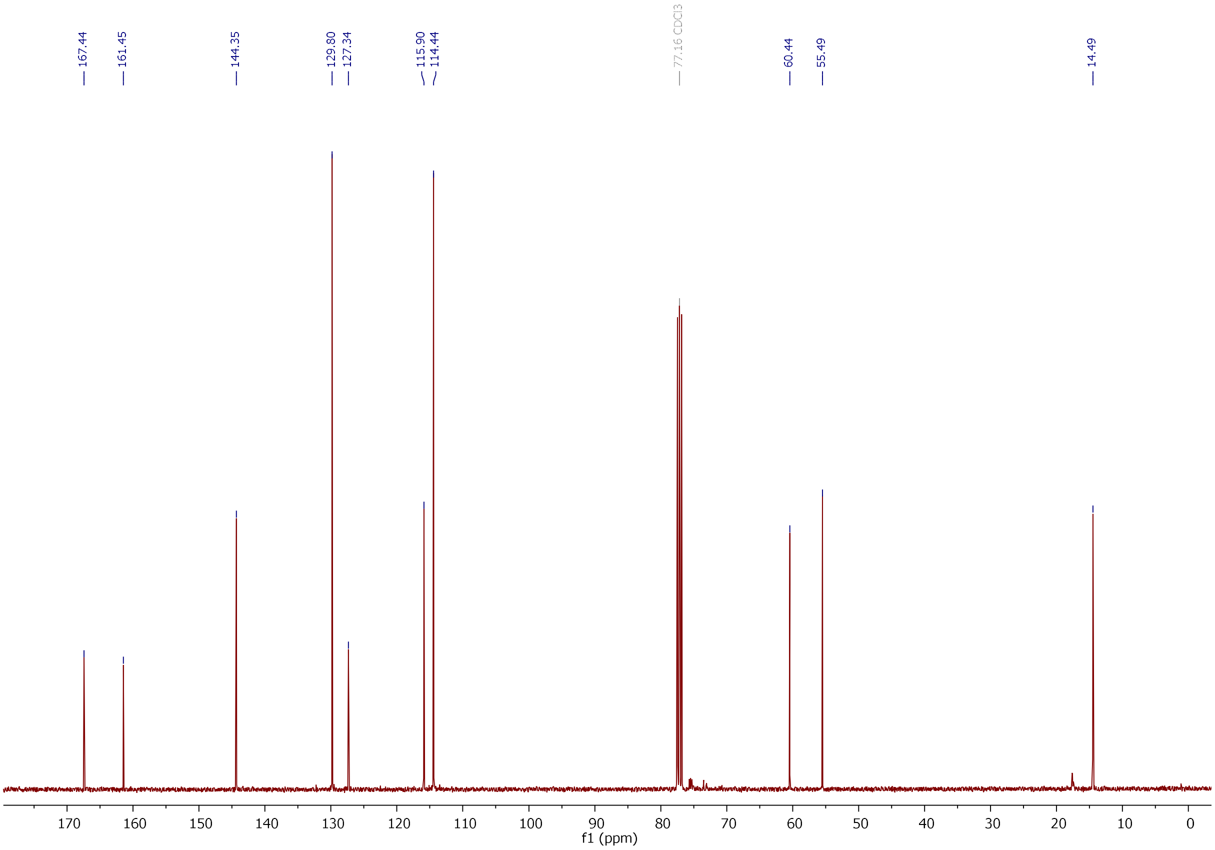

(*E*)-(4-methoxystyryl)trimethylsilane (4b)

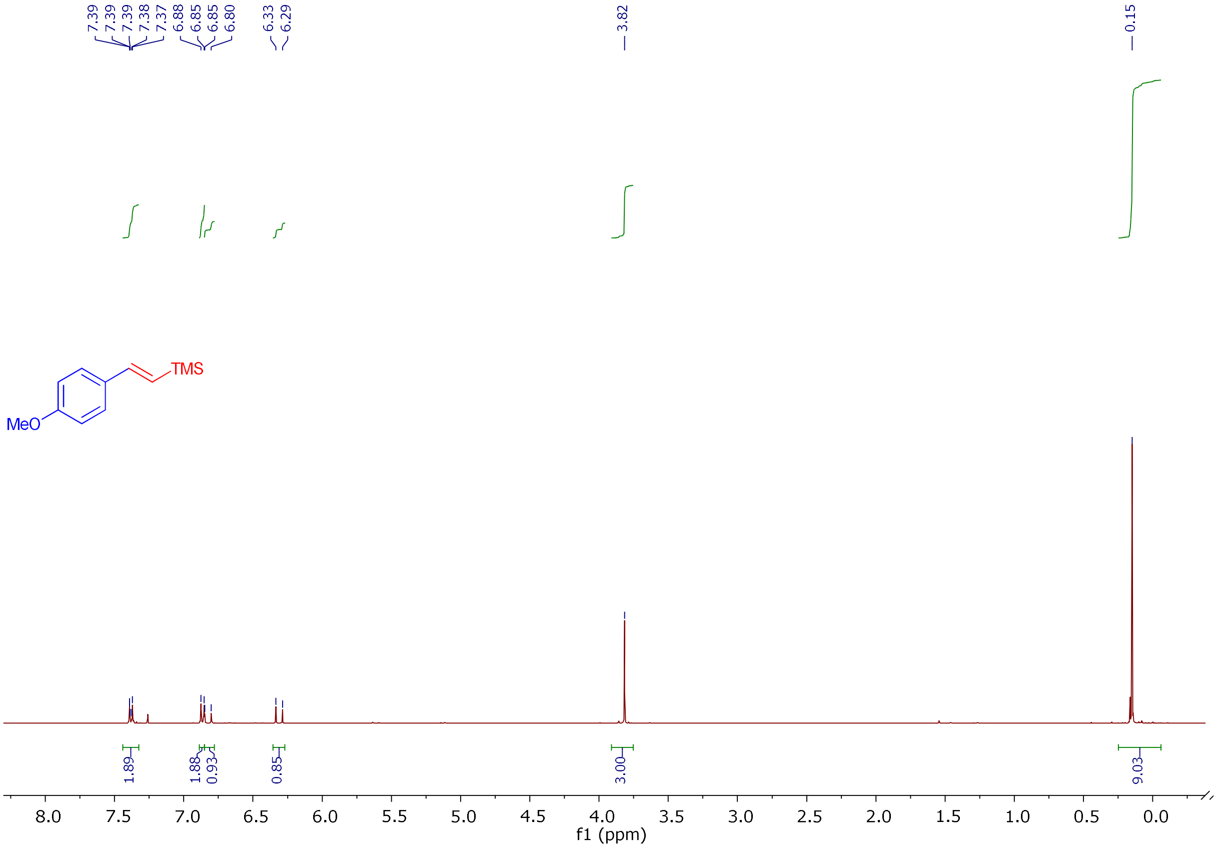

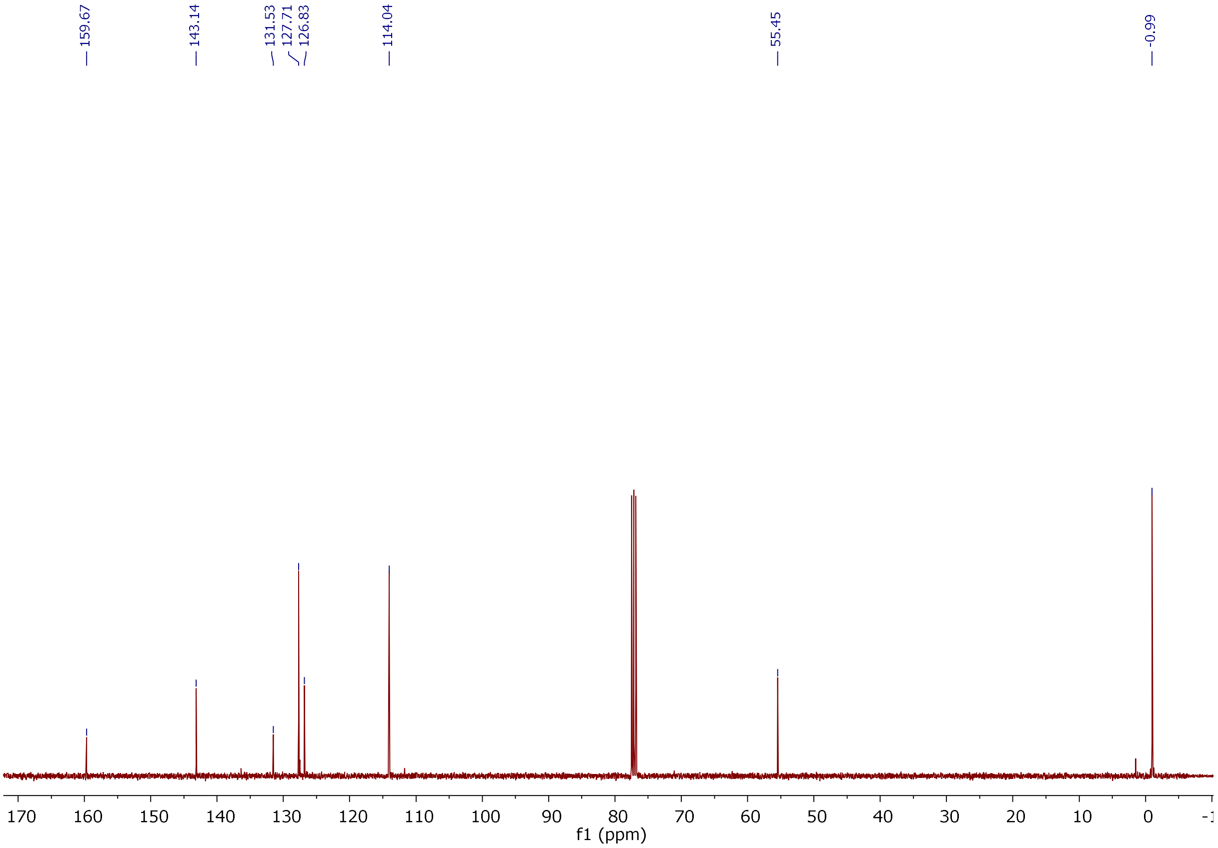

(*E*)-Ethyl 3-(3,4-dimethoxyphenyl)acrylate (4c)

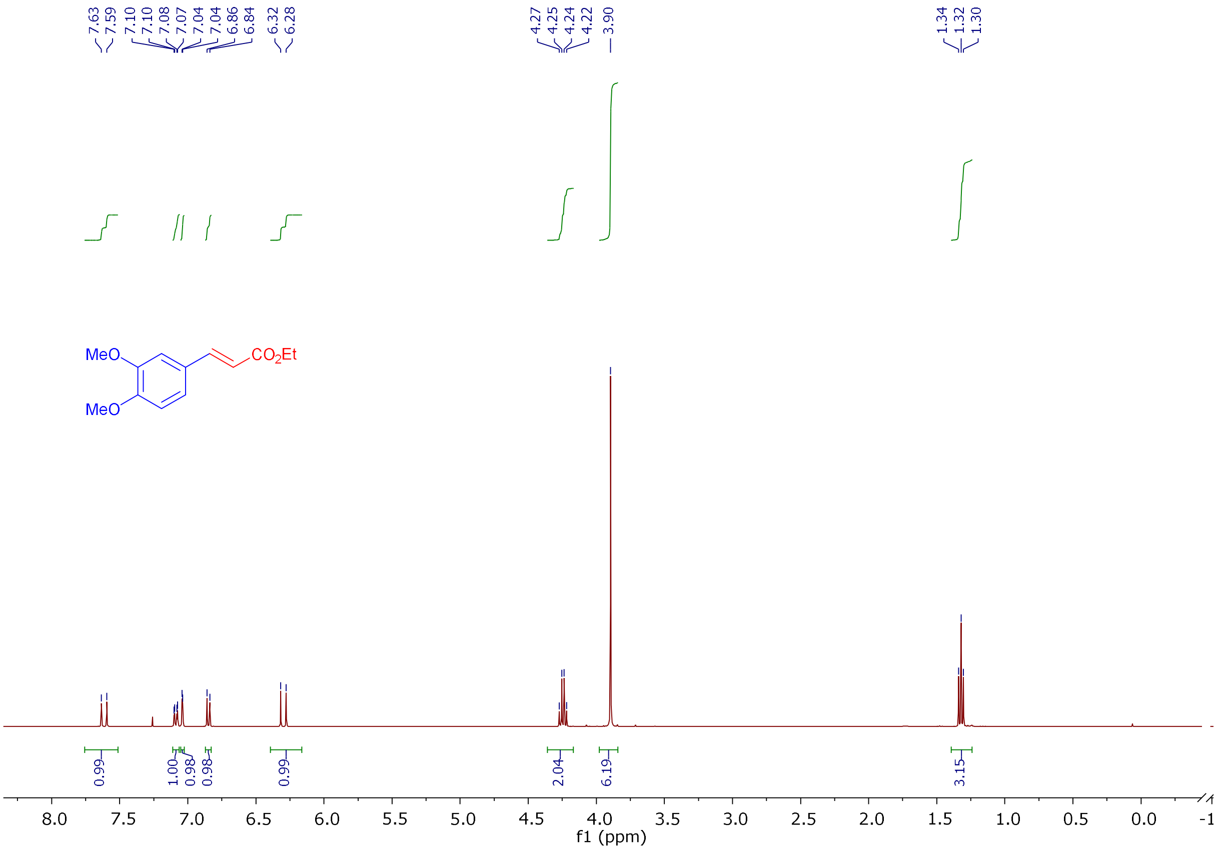

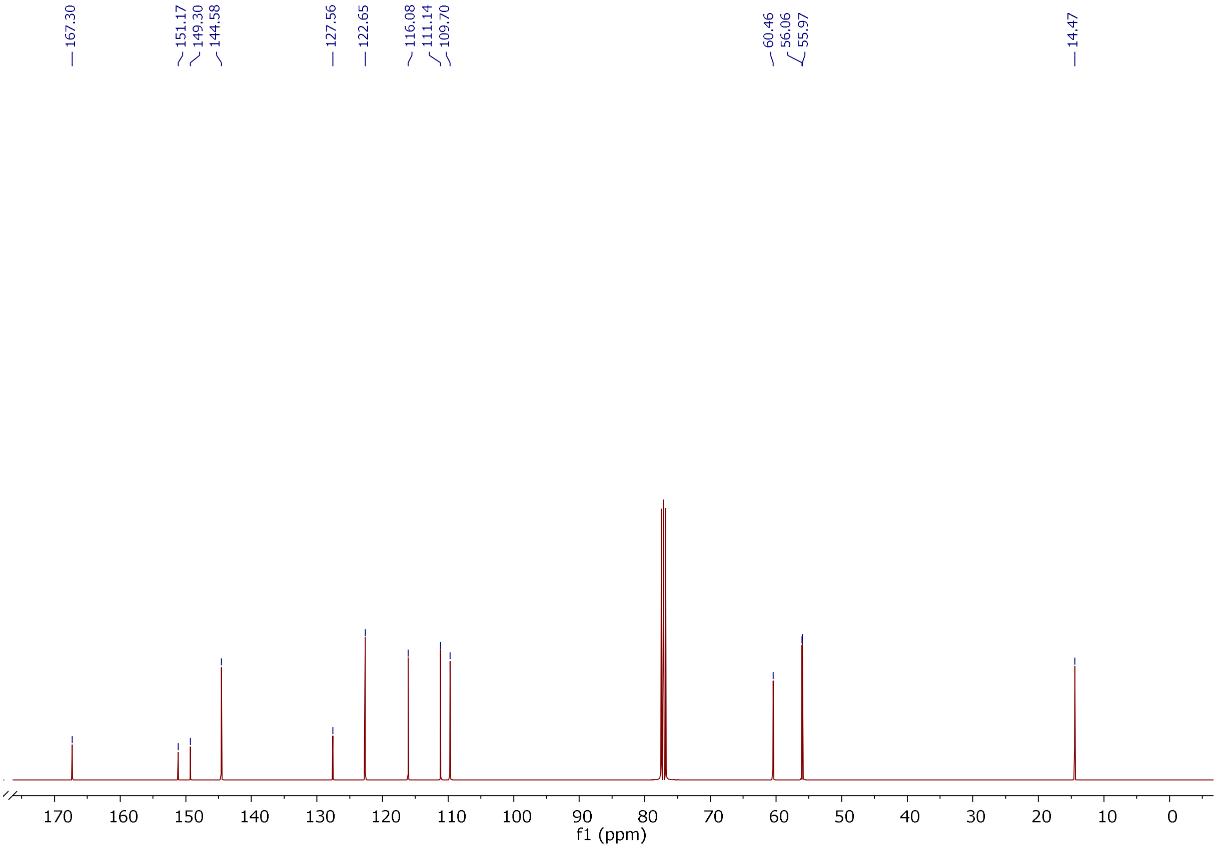

(*E*)-Trimethyl(3,4,5-trimethoxystyryl)silane (4d)

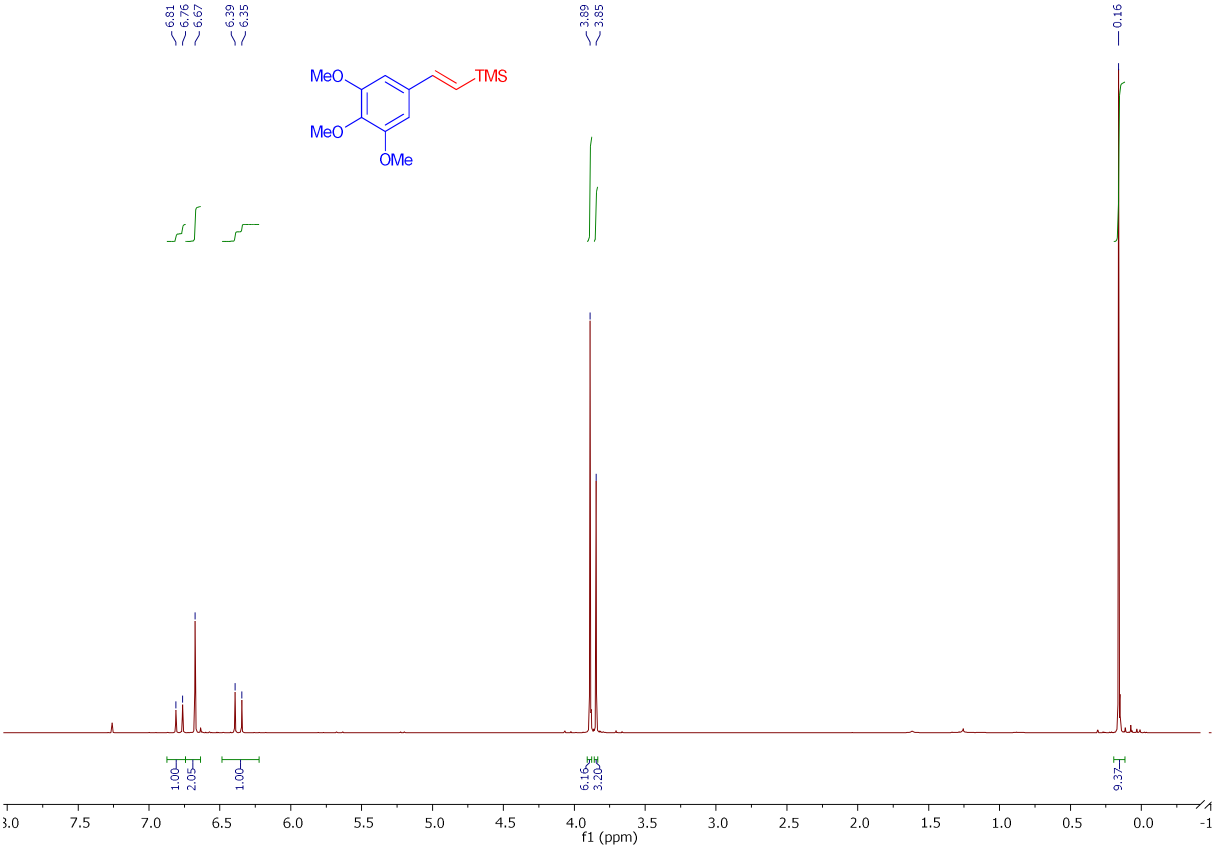

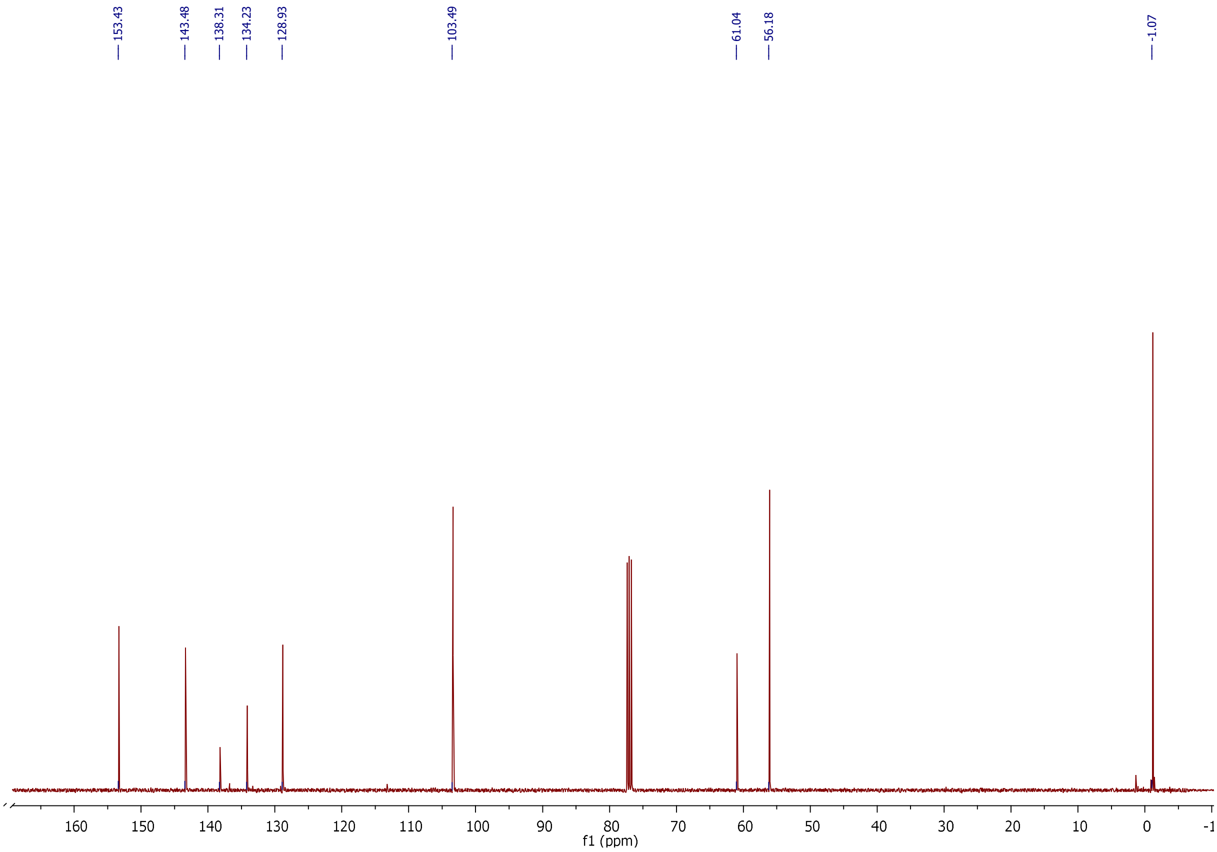

(*E*)-Ethyl 3-(3,4,5-trimethoxyphenyl)acrylate (4e)

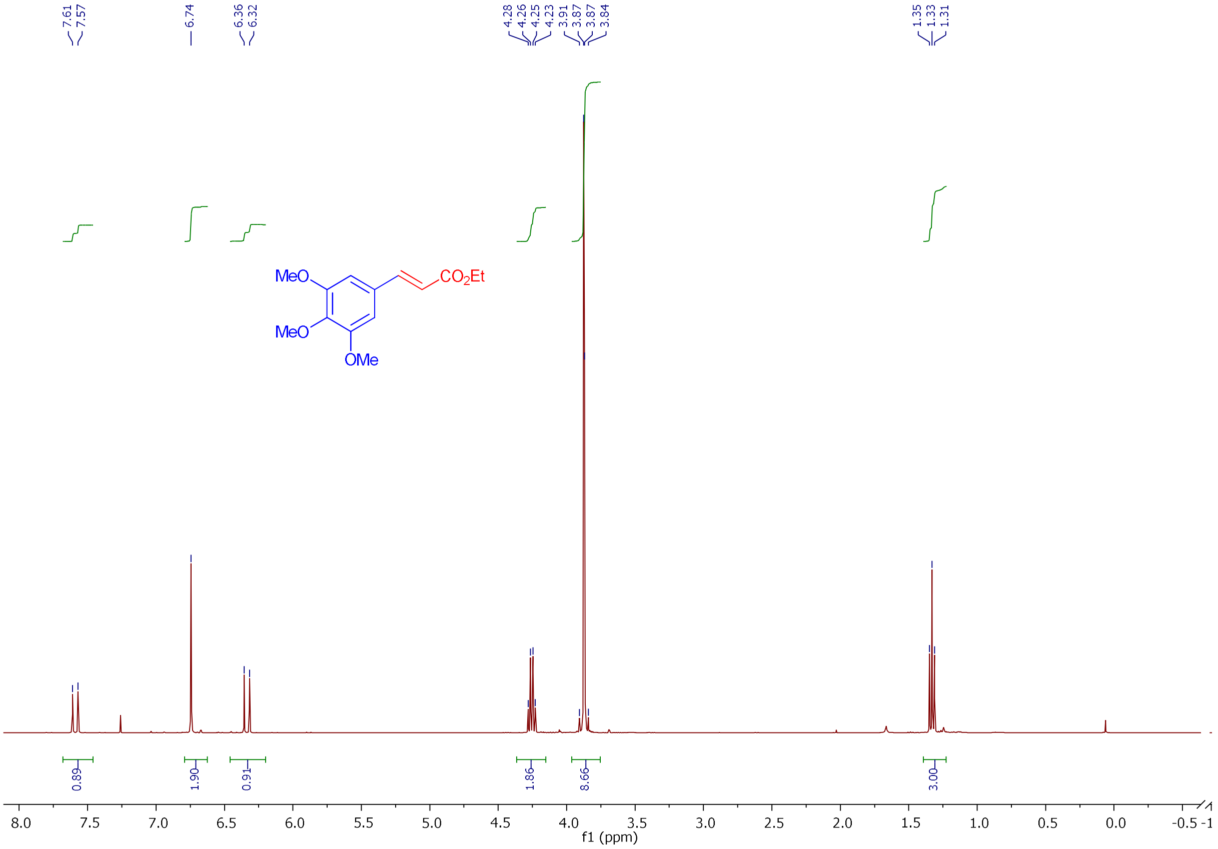

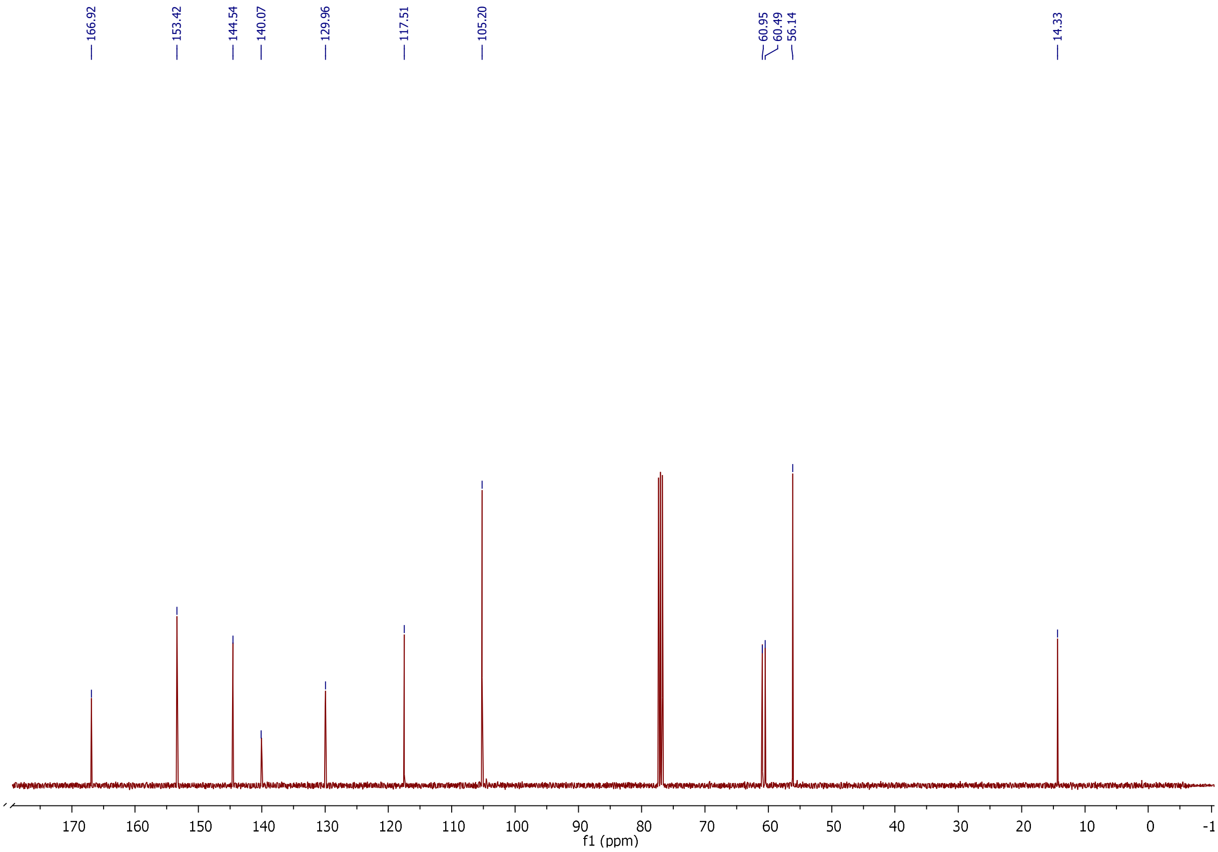

(*E*)-1,2,3-trimethoxy-5-styrylbenzene (4f)

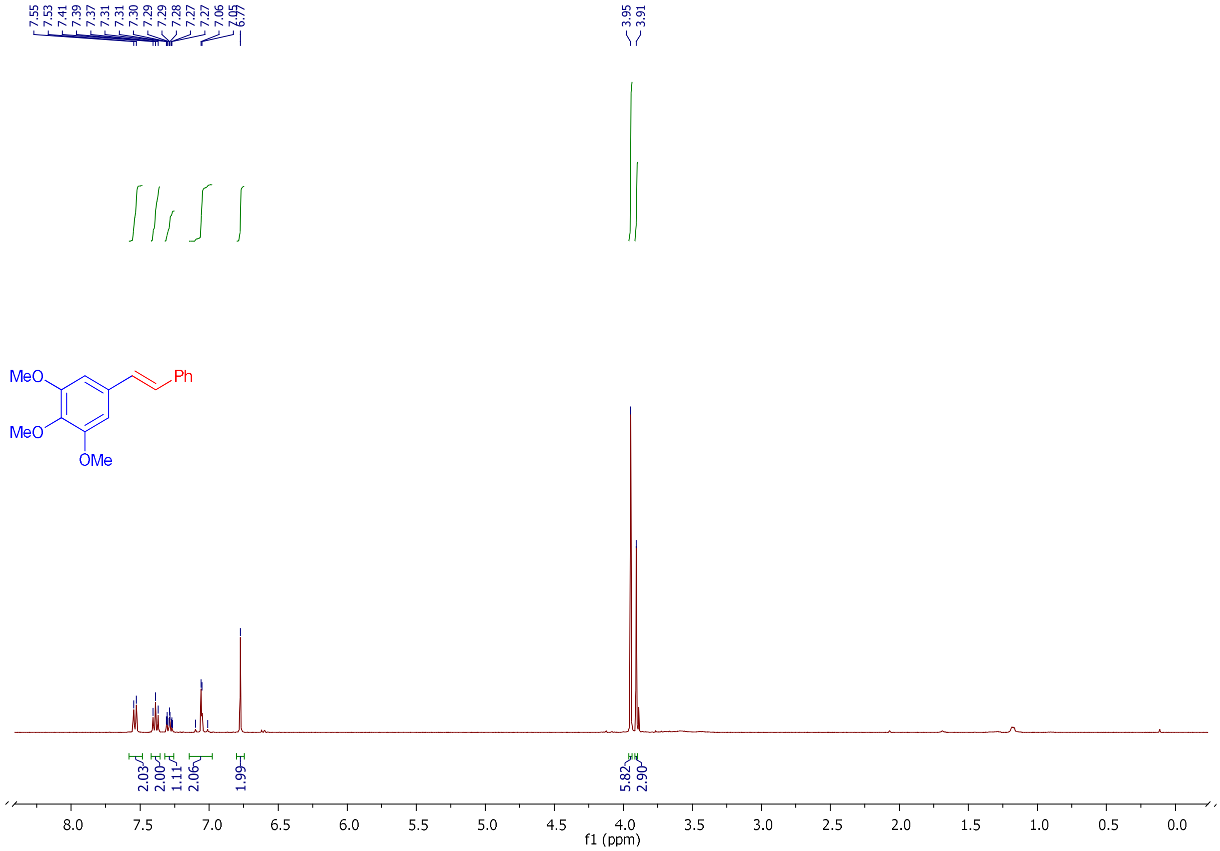

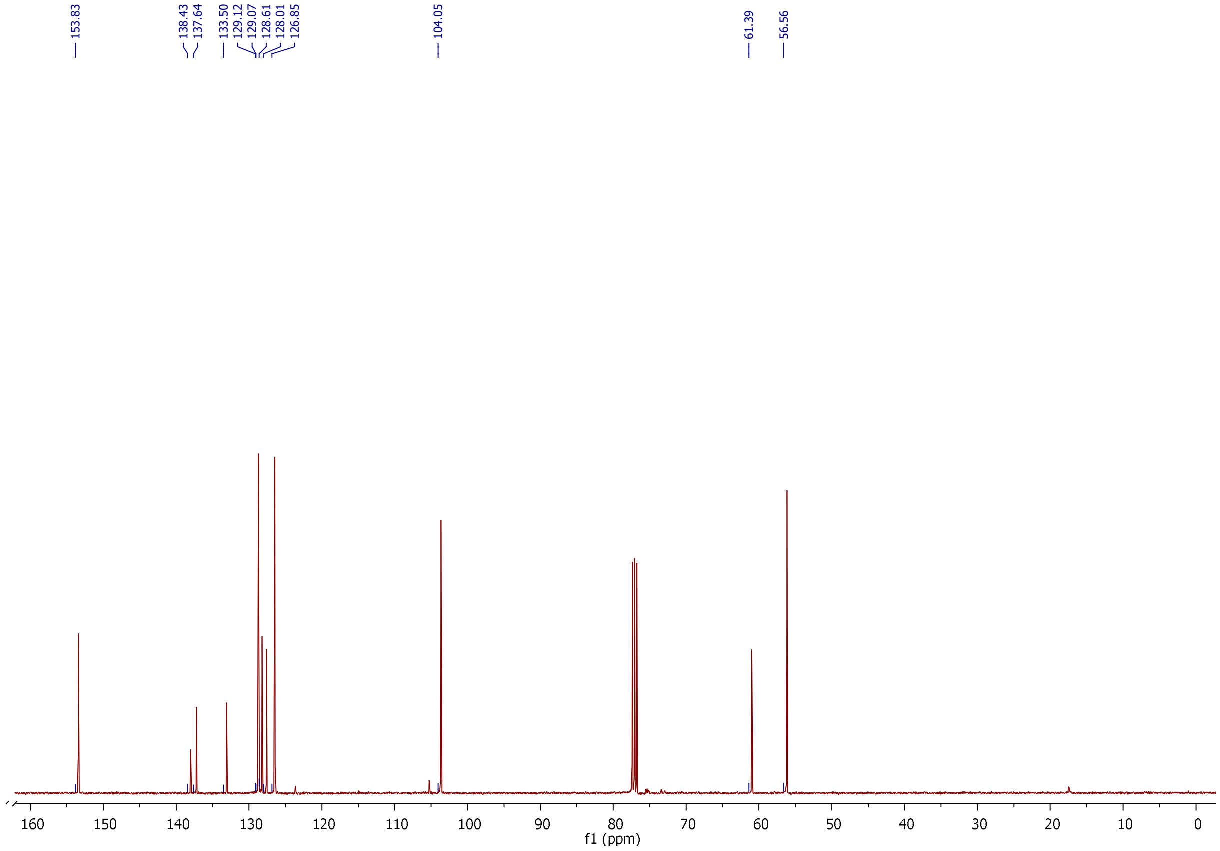

1,2,3-Trimethoxy-5-(oct-1-en-1-yl)benzene (4g)

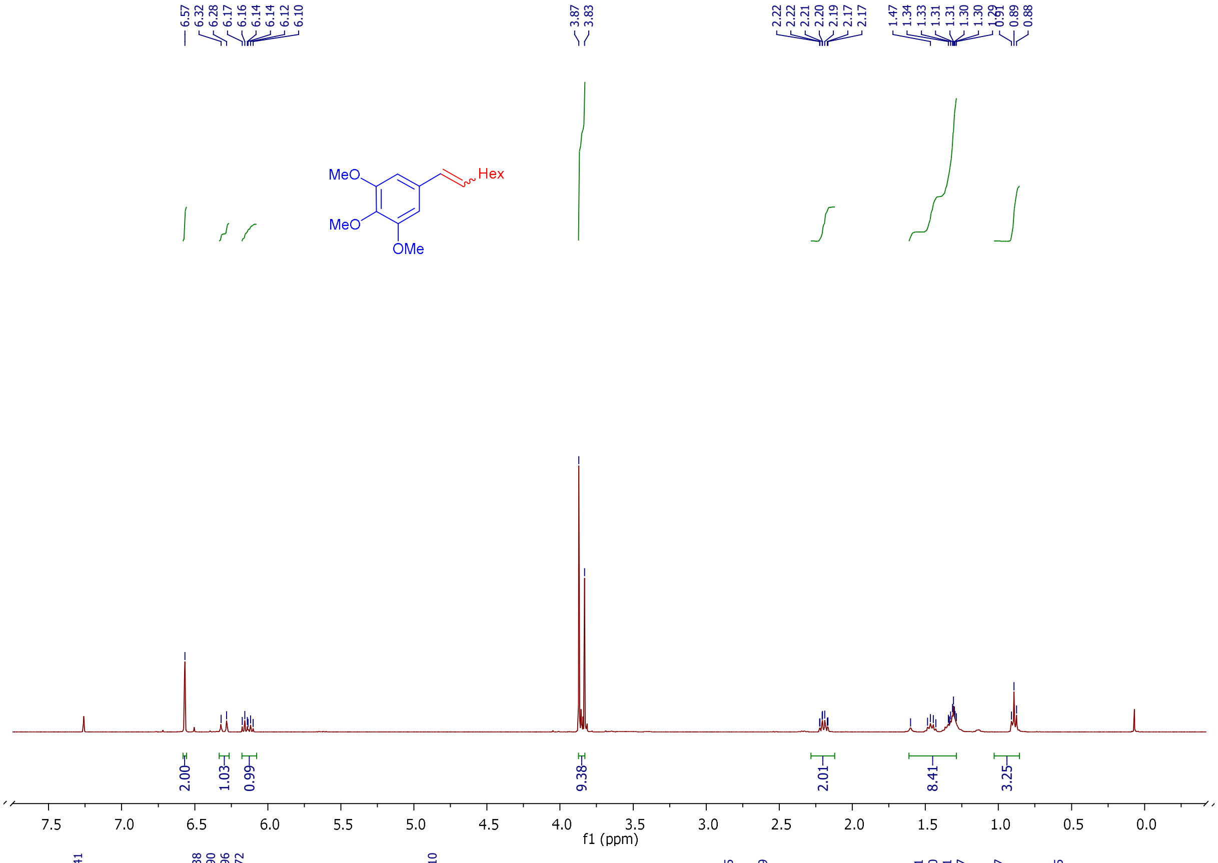

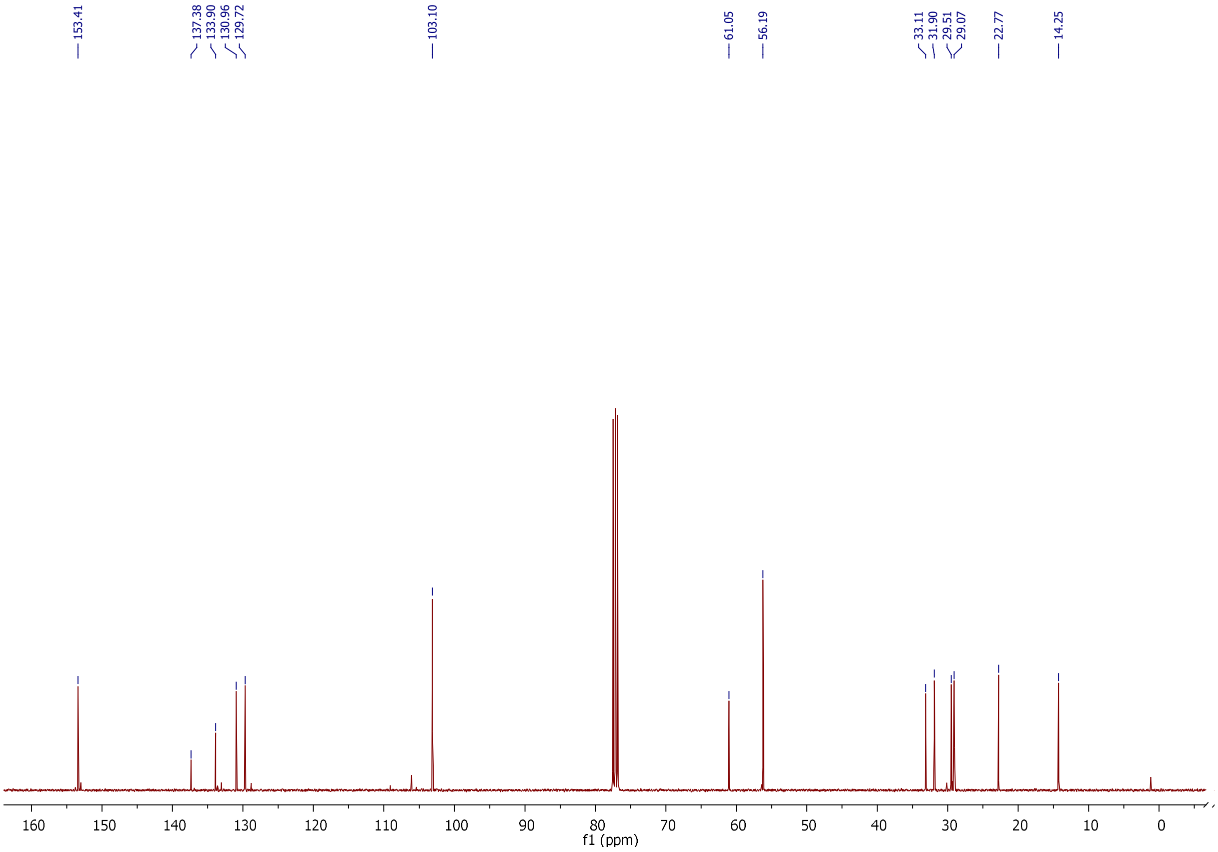

(*E*)-Ethyl 3-(4-(trifluoromethoxy)phenyl)acrylate (4h)

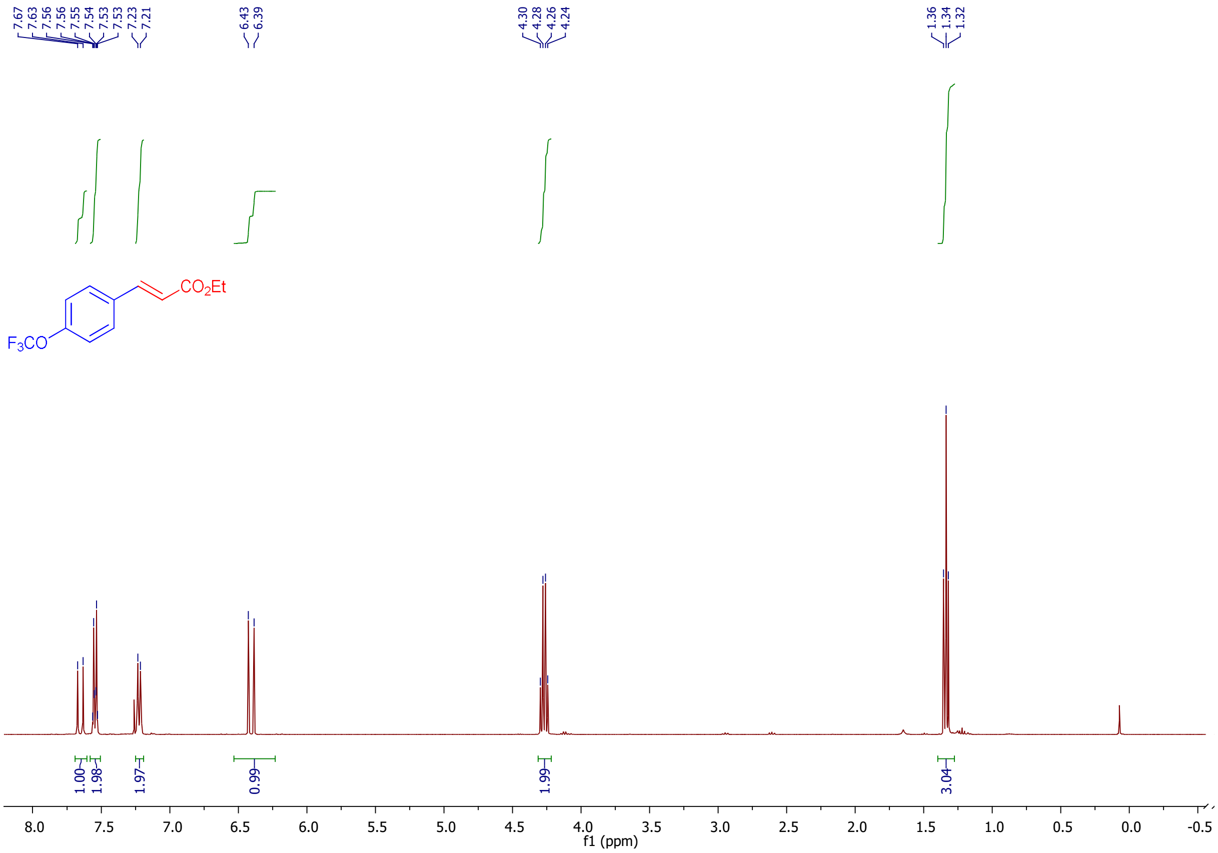

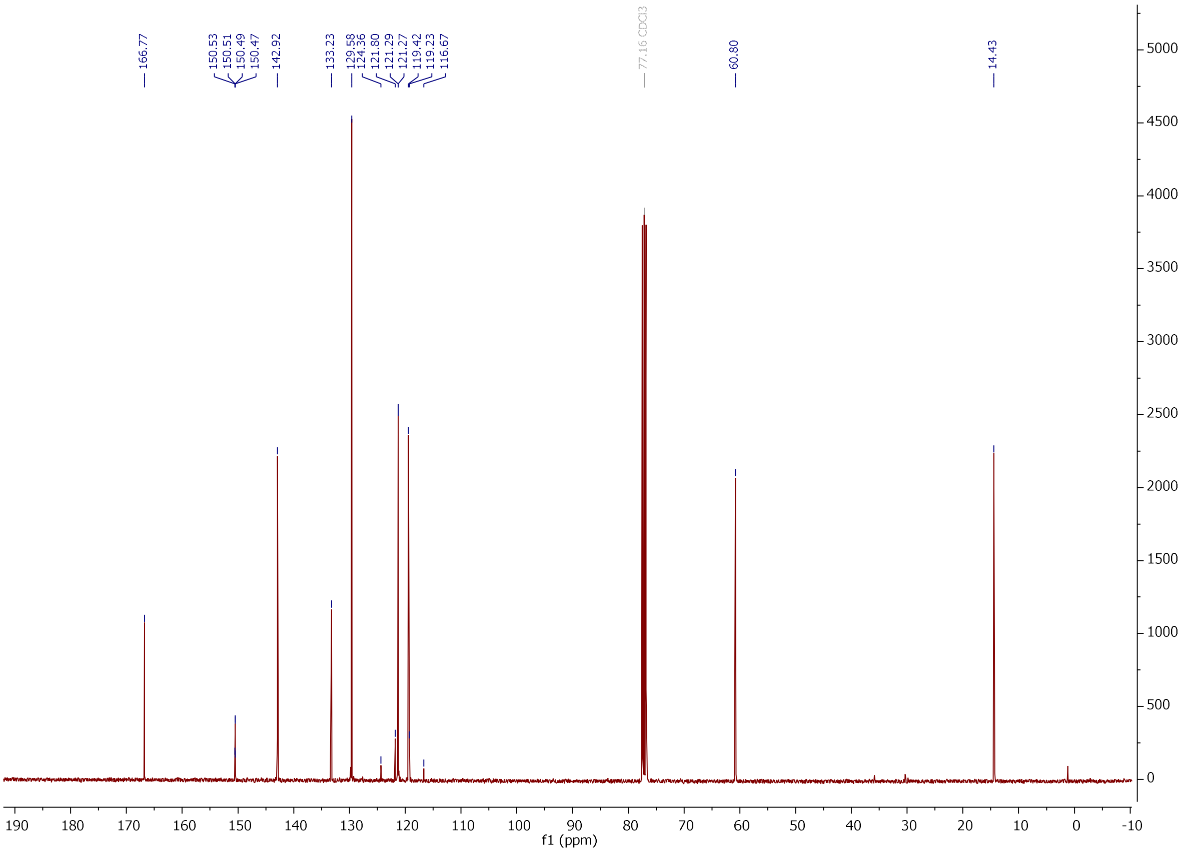

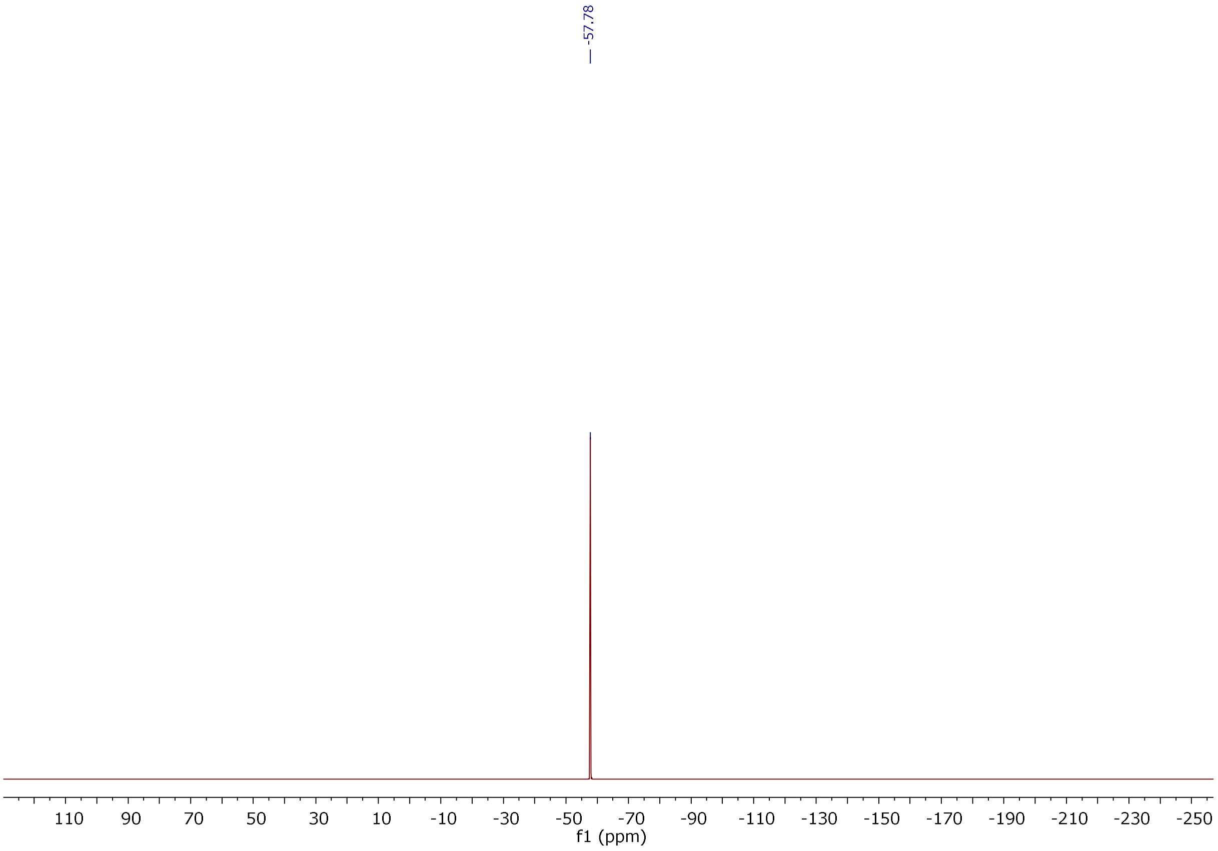

(*E*)-Ethyl 3-(3-(trimethylsilyl)phenyl)acrylate (4i)

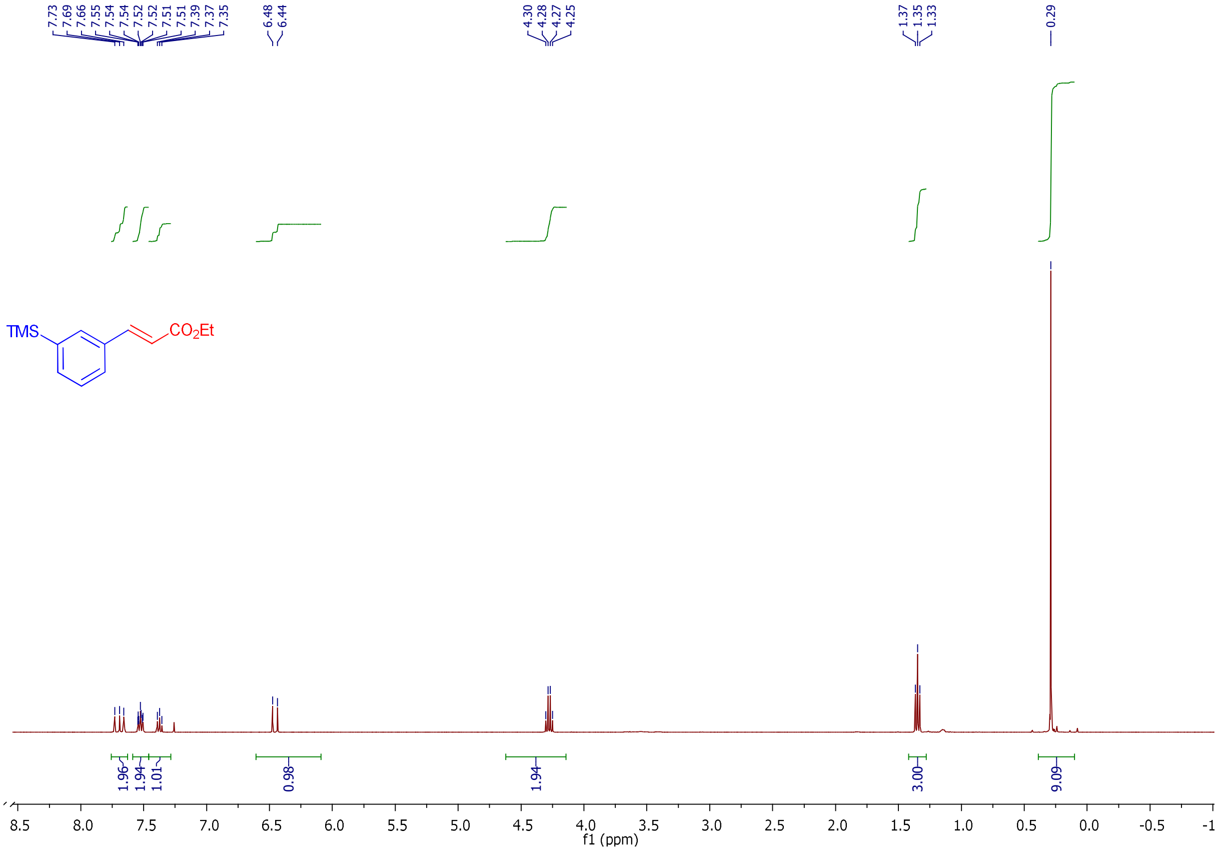

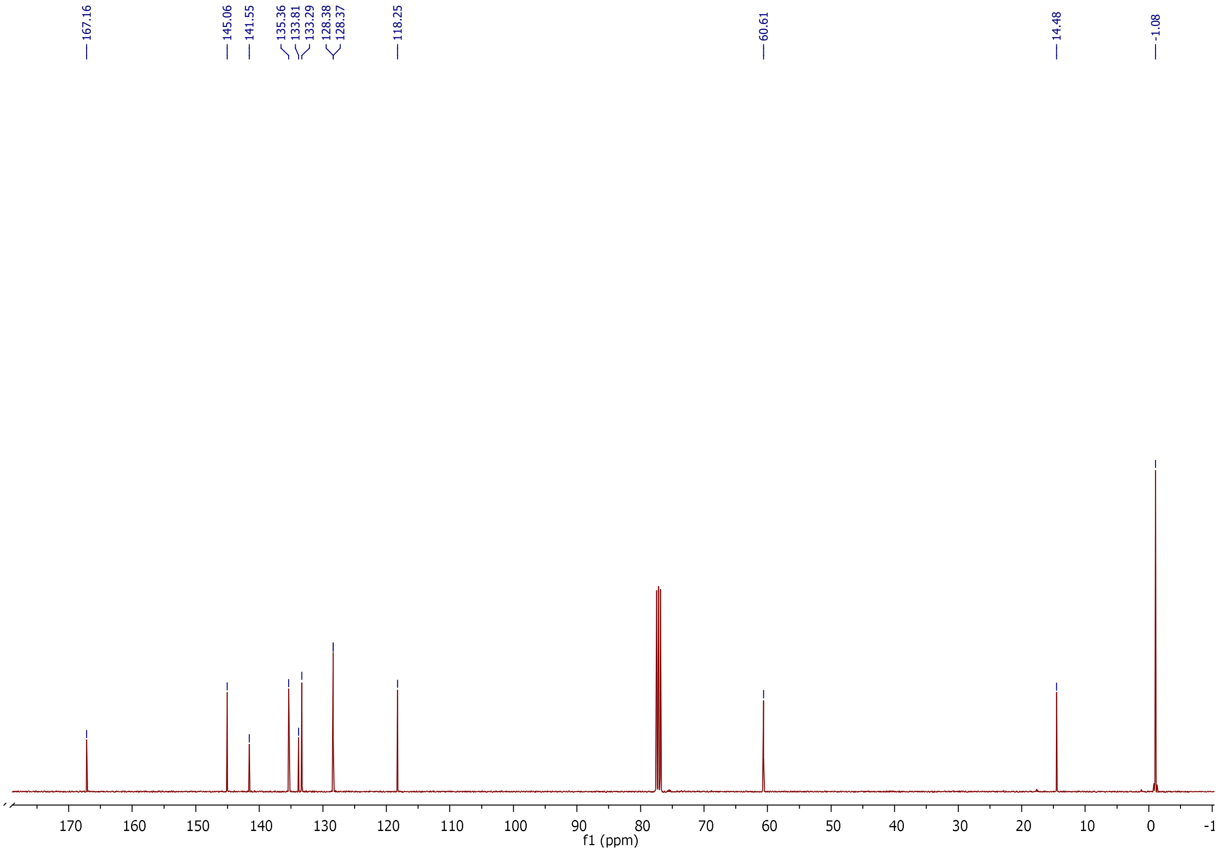

(*E*)-(2-(benzo[d][1,3]dioxol-5-yl)vinyl)trimethylsilane (4j)

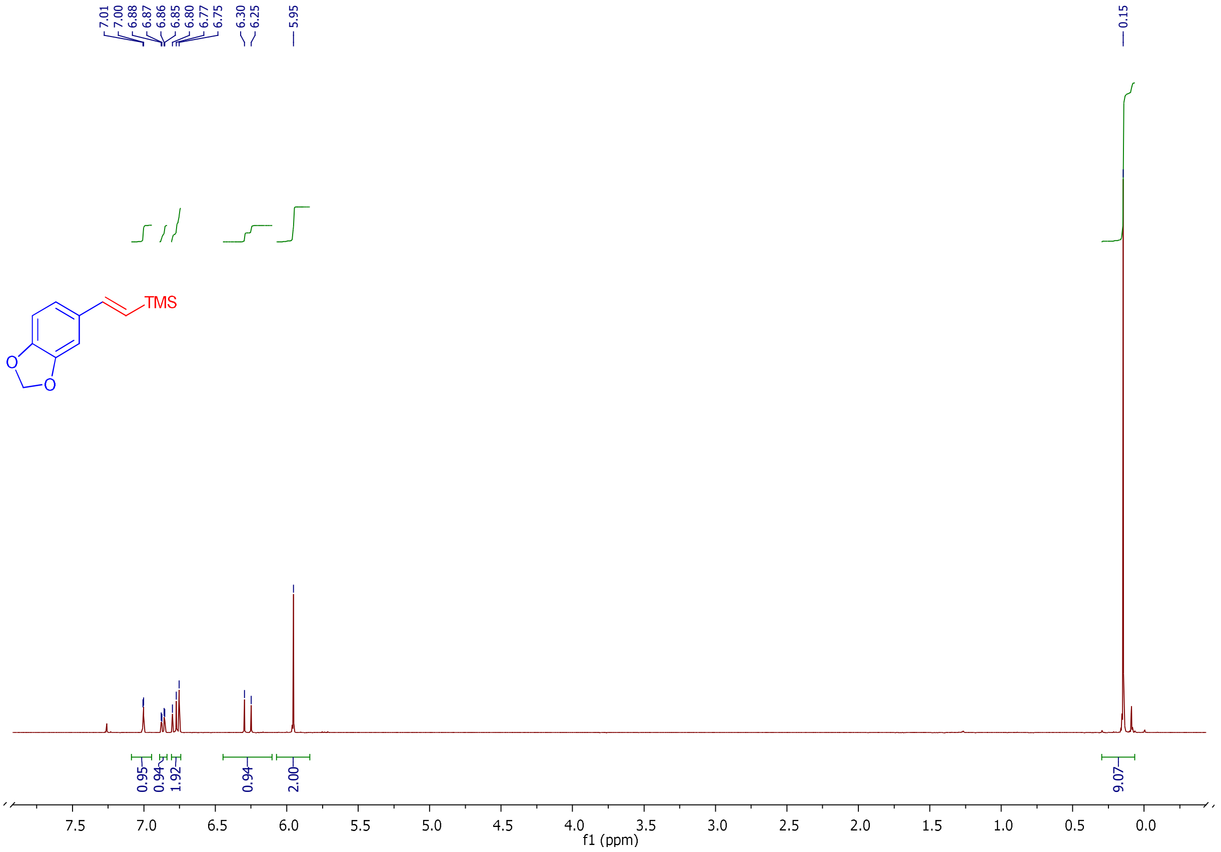

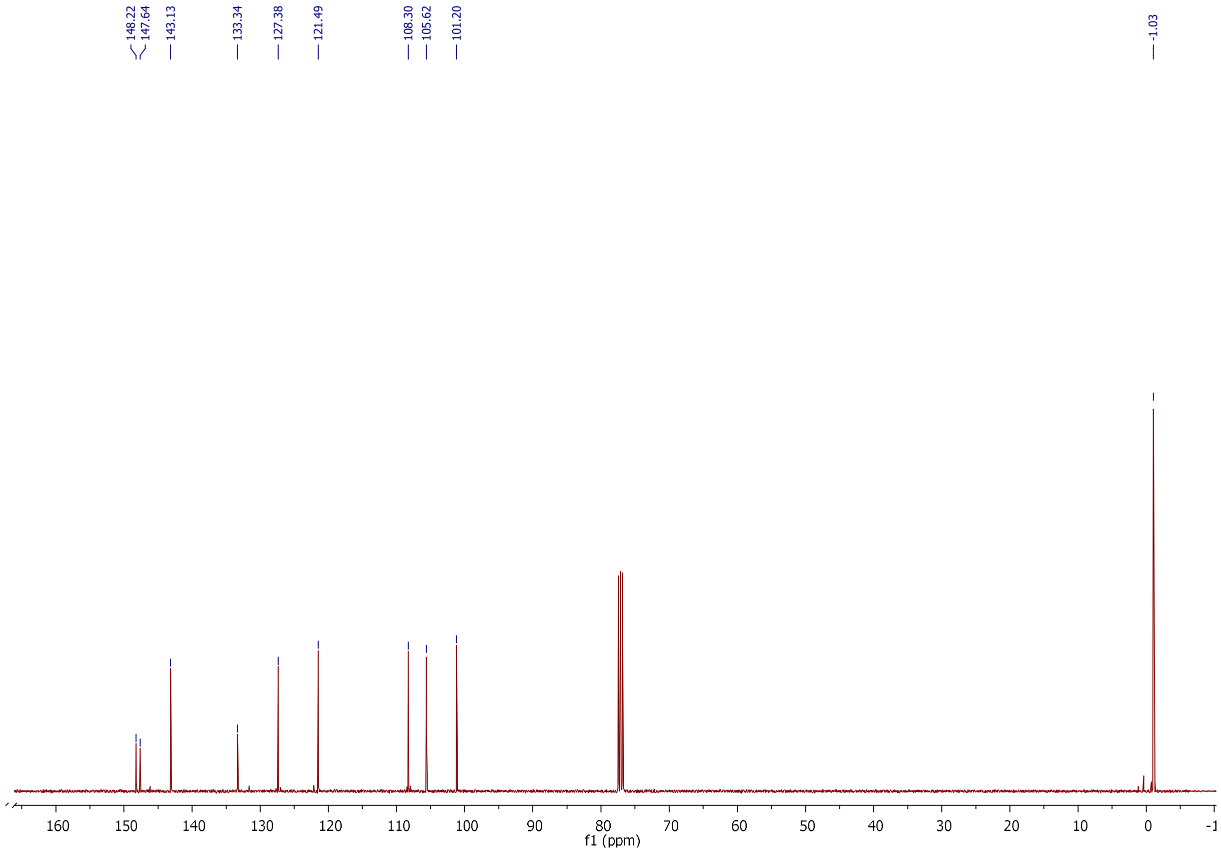

(*E*)-Ethyl 3-(benzo[d][1,3]dioxol-5-yl)acrylate (4k)

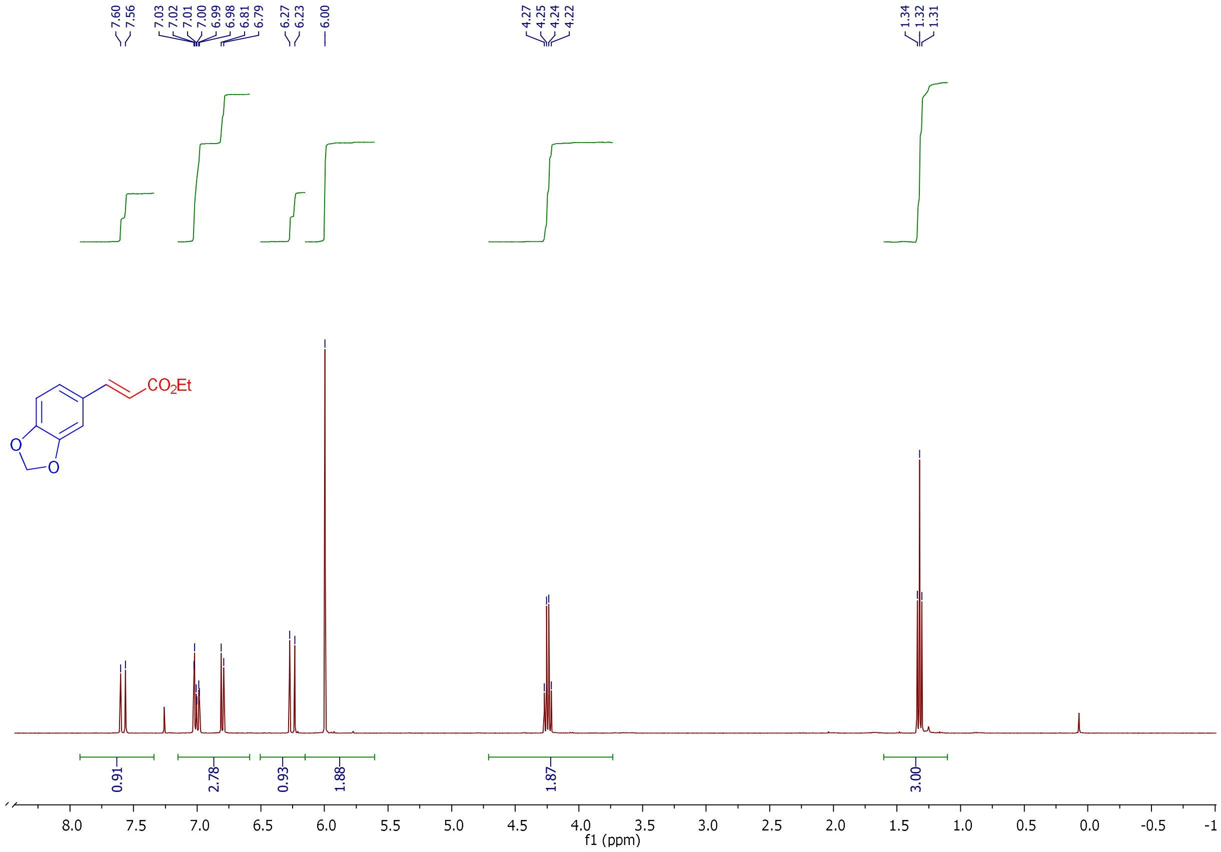

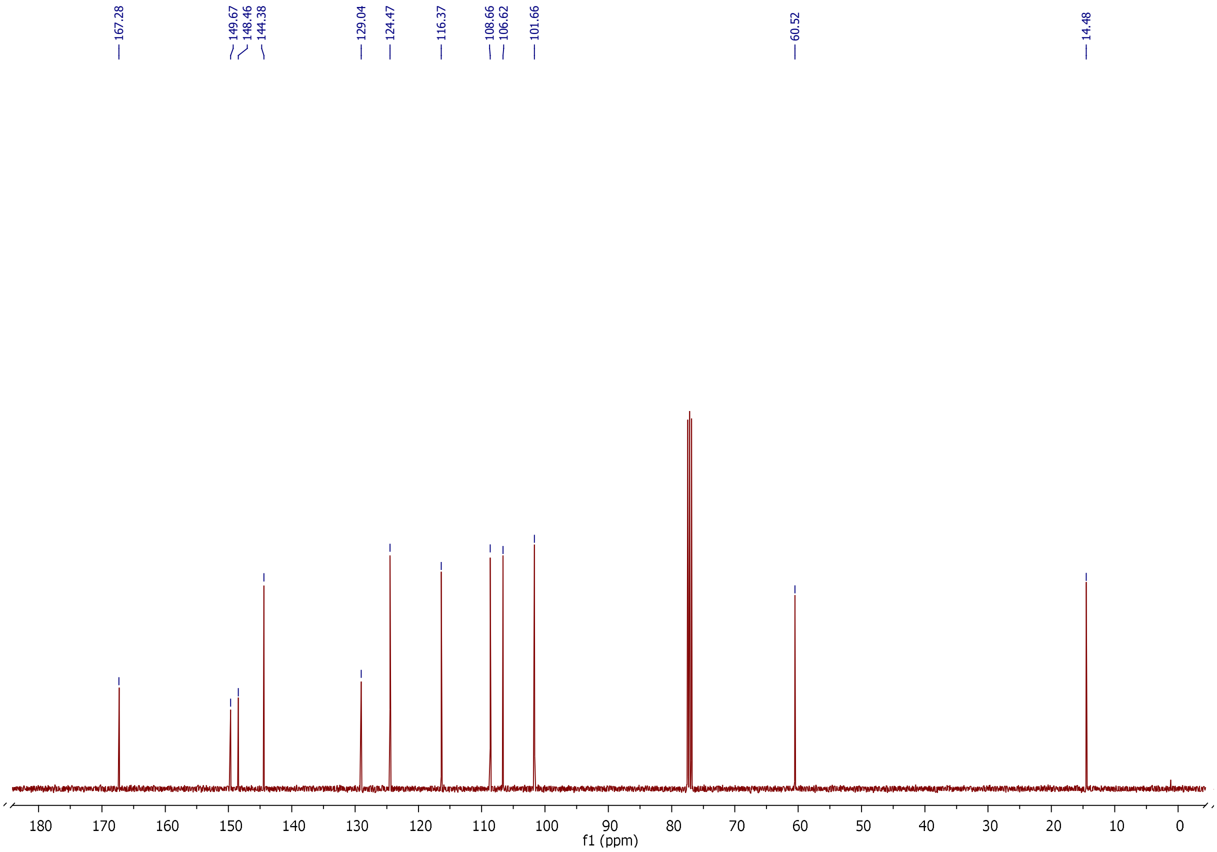

(*Z*)-4-(4-(trifluoromethoxy)styryl)benzonitrile (4m)

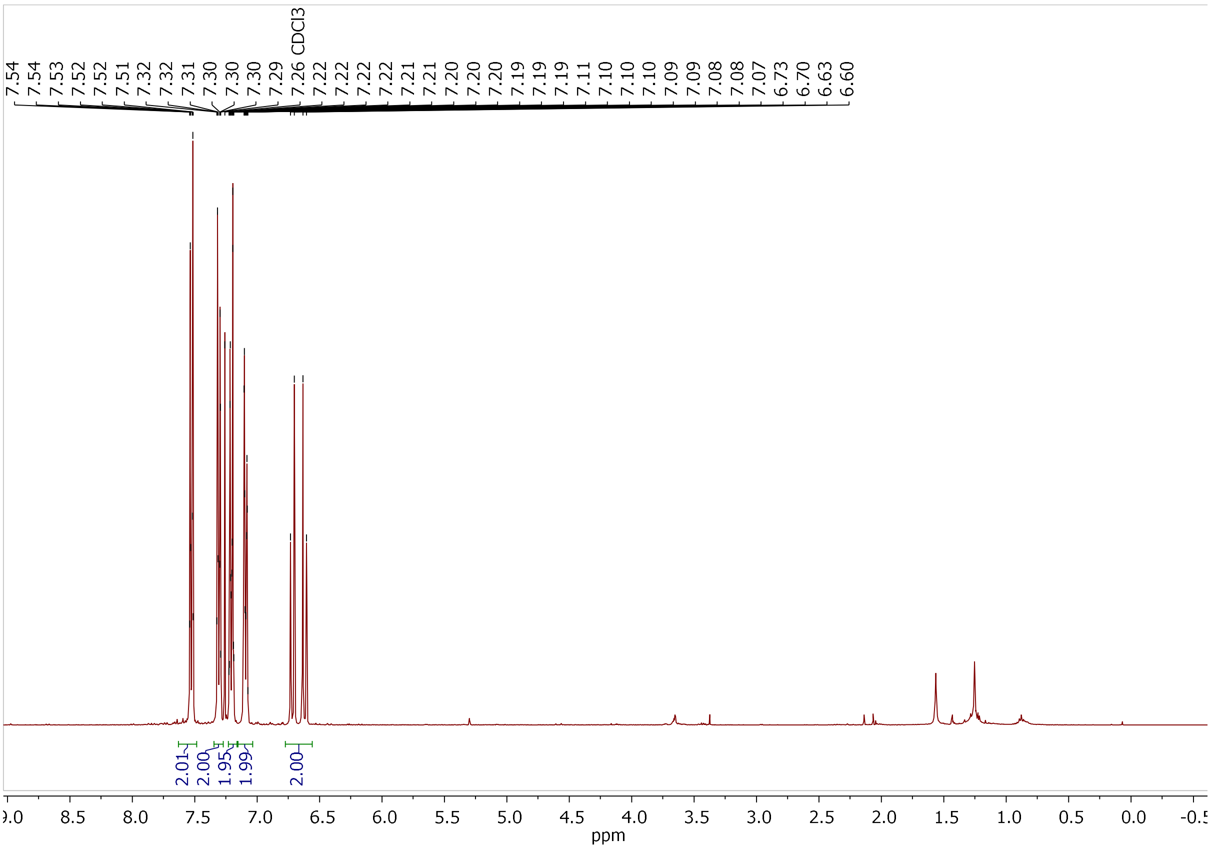

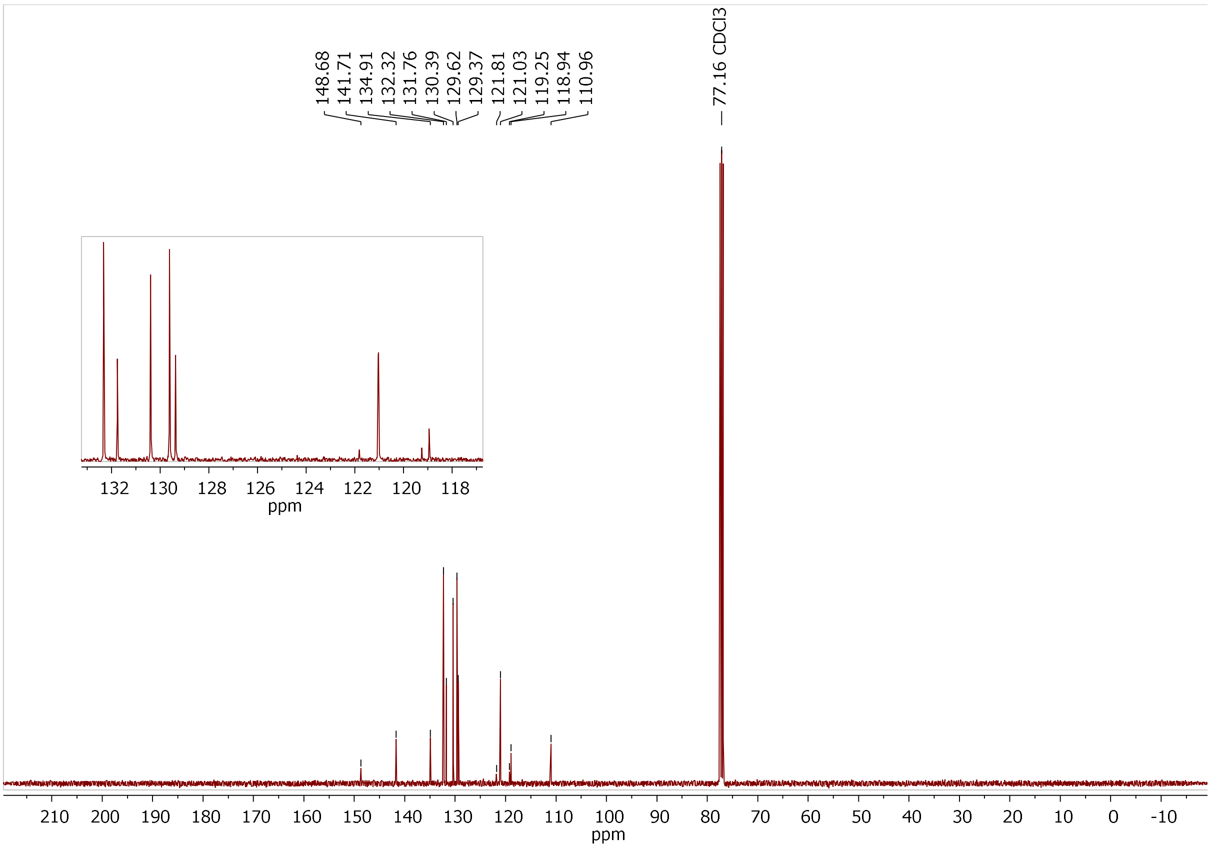
148.68
141.71
134.91
132.32
131.76
130.39
129.62
129.37
121.81
121.03
119.25
118.94
110.96
77.16 CDCl3
ppm
ppm

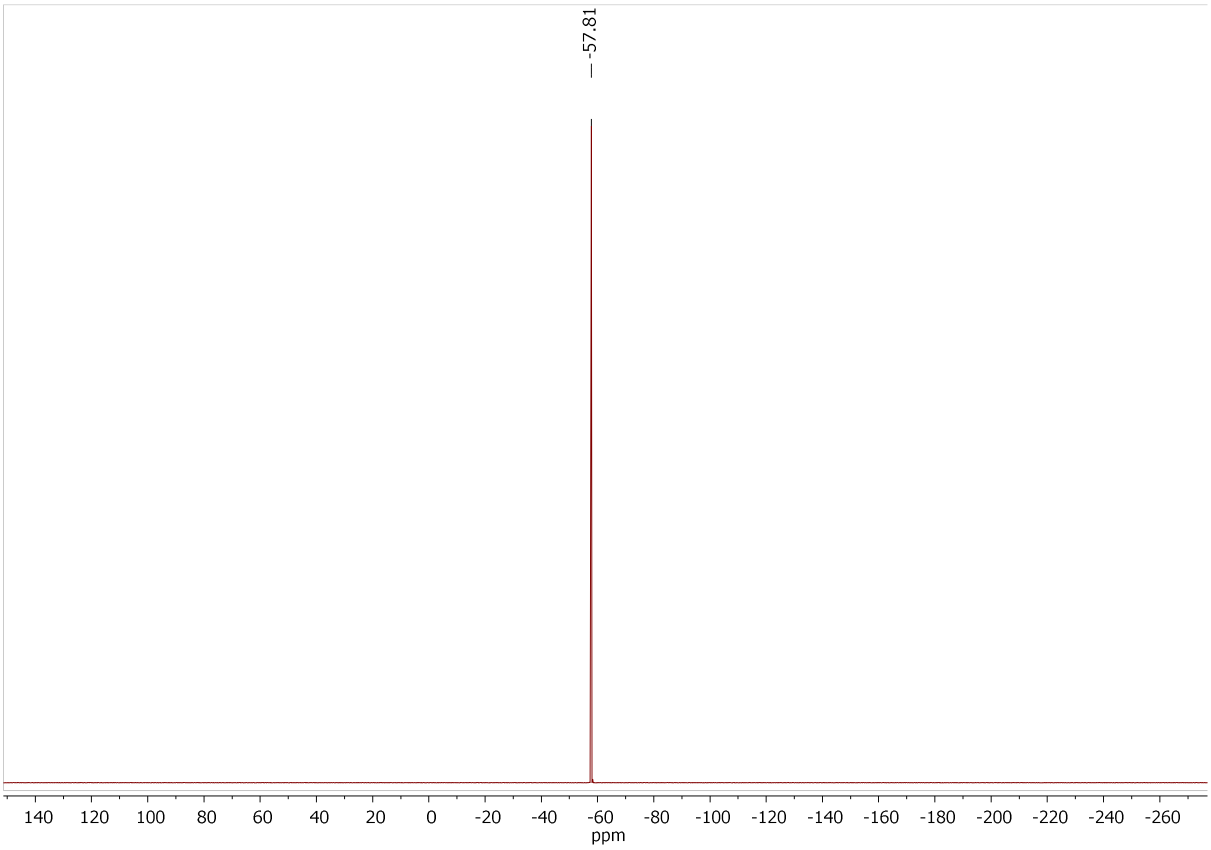
-57.81
ppm

Synthesis of 4-(3-(trimethylsilyl)styryl)benzonitrile (4n)

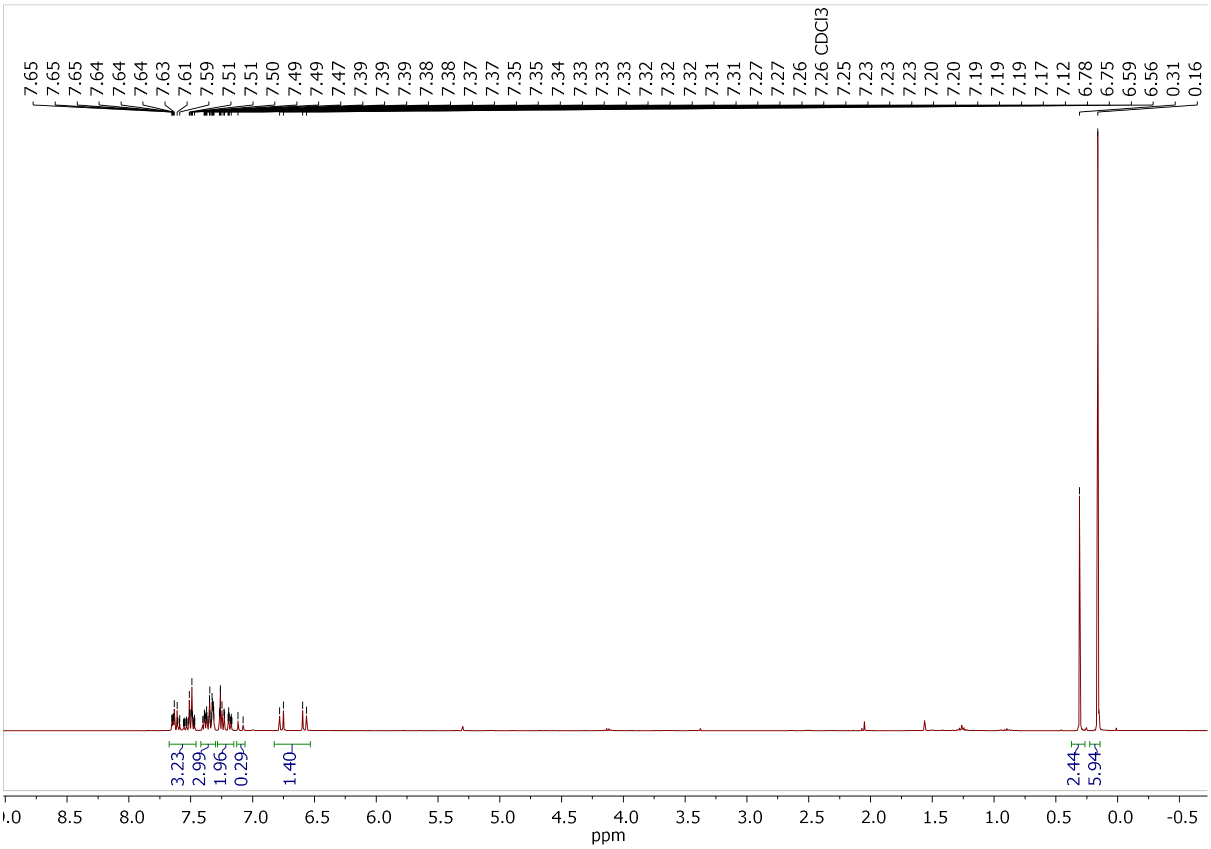

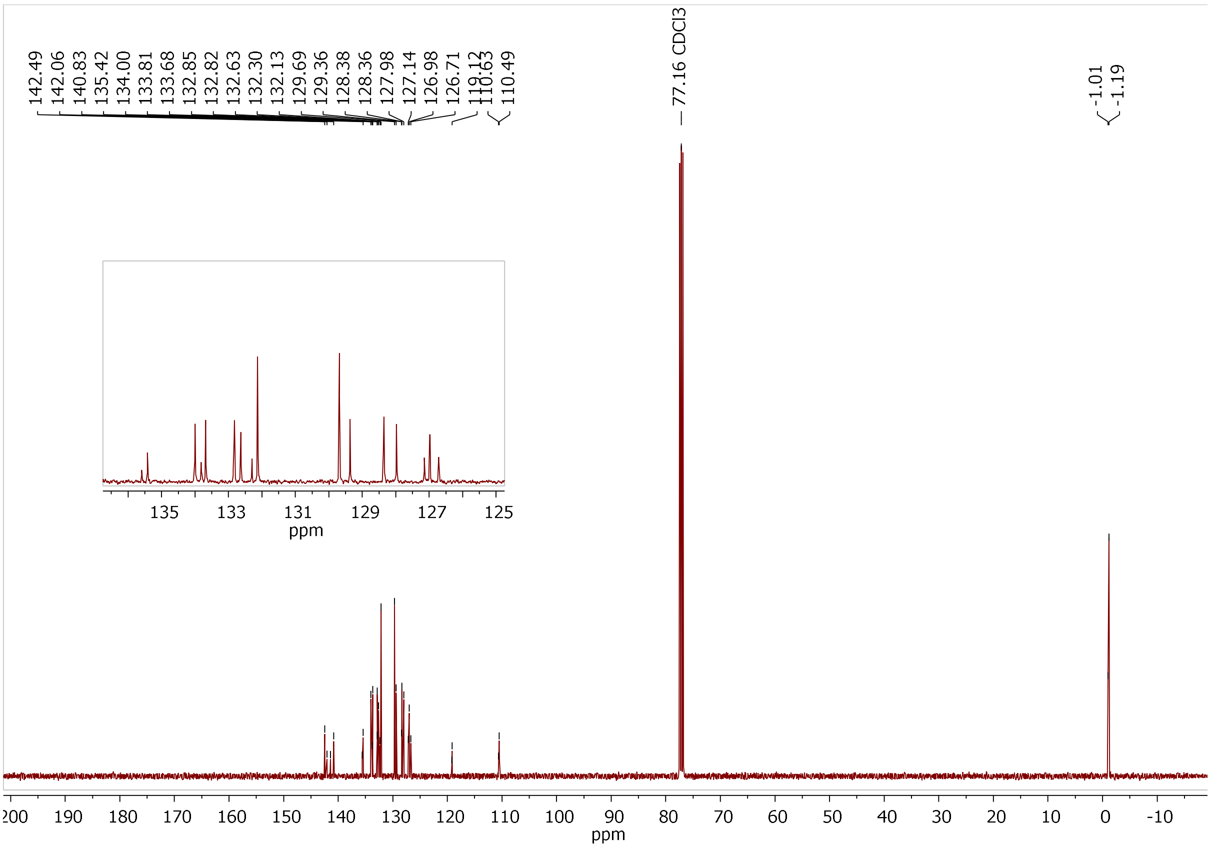

Supplement: Supplementary file 1 [file molecules-25-00723-s001.pdf]
